# Implementing complex nucleic acid circuits in living cells

**DOI:** 10.1126/sciadv.adv6512

**Published:** 2025-04-30

**Authors:** Jiajia Sun, Xiewei Xiong, Wei Lai, Zhongdong Wu, Heming Wang, Lei Yang, Niannian Xue, Qunyan Yao, Guangqi Song, Yicheng Zhao, Li Li, Fei Wang, Chunhai Fan, Hao Pei

**Affiliations:** ^1^ Shanghai Key Laboratory of Green Chemistry and Chemical Processes, School of Chemistry and Molecular Engineering, Shanghai Center of Brain-inspired Intelligent Materials and Devices, East China Normal University, Shanghai 200241, China.; ^2^ Hubei Key Laboratory of Energy Storage and Power Battery, School of Mathematics, Physics and Optoelectronic Engineering, Hubei University of Automotive Technology, Shiyan, Hubei 442002, China.; ^3^ Joint Laboratory of Biomaterials and Translational Medicine, Puheng Biomedicine Co. Ltd, Shanghai 201203, China.; ^4^ Shanghai Frontiers Science Center of Genome Editing and Cell Therapy, Shanghai Key Laboratory of Regulatory Biology, Institute of Biomedical Sciences and School of Life Sciences, East China Normal University, Shanghai 200241, China.; ^5^ Department of Gastroenterology and Hepatology, Zhongshan Hospital, Fudan University, Shanghai 200032, China.; ^6^ Chinese Medicine Guangdong Laboratory, Hengqin, Guangdong 519031, China.; ^7^ School of Chemistry and Chemical Engineering, New Cornerstone Science Laboratory, Frontiers Science Center for Transformative Molecules, National Center for Translational Medicine, Shanghai Jiao Tong University, Shanghai 200240, China.

## Abstract

Synthetic nucleic acid–based computing has demonstrated complex computational capabilities in vitro. However, translating these circuits into living cells remains challenging because of instability and cellular interference. We introduce an allosteric strand exchange (ASE) strategy for complex intracellular computing. Leveraging conformational cooperativity to regulate strand exchange, ASE offers a modular platform for designing intracellular circuits with flexible programmability. We engineer a scalable circuit architecture based on ASE that can execute AND and OR logic and scale to an eight-input expression. We demonstrate ASE-based circuits can detect messenger RNAs with high specificity in mammalian cells via AND logic computation. The capacity of ASE-based circuits to accept messenger RNAs as inputs enables integration of endogenous cellular information for efficient multi-input information processing, demonstrated by a multi-input molecular classifier monitoring key cell reprogramming events. Reprogramming ASE-based circuit to interface with CRISPR-Cas9 enables programmable control of Cas9-targeting activity for gene editing, highlighting their potential for advancing intracellular biocomputation.

## Teaser

Allosteric strand exchange enables the implementation of scalable nucleic acid–based computing in mammalian cells.

## INTRODUCTION

Nucleic acid computing has emerged as a transformative approach for molecular information processing directly in chemical and biological contexts, demonstrating substantial potential in applications such as molecular diagnostics, data storage, and information security (*1*–*6*). Increasingly sophisticated computational molecular circuits, based on toehold-mediated three-way strand displacement mechanisms (*7*–*11*), have been realized in test tubes to perform a range of highly complex tasks, including digital logic (*8*, *12*–*17*), nonlinear dynamics (*18*–*21*), pattern recognition (*22*–*24*) and decision-making (*25*, *26*). These multilayered circuits typically use single-stranded oligonucleotides as the uniform input/output signals, enabling the cascading of multiple strand displacement reactions to create intricate molecular systems. These advancements highlight the potential of synthetic nucleic acids for fully de novo design of diverse functional components, circuits, and systems for application purposes, with the ability to increase circuit size in a modular manner (*27*–*31*). Despite the remarkable progress for studies in cell-free settings, there remain enormous challenges to develop these complex nucleic acid–based computing devices in living cells, which is crucial for fully realizing their potential in biology applications (*1*, *4*, *27*, *32*). Given the complex intracellular environment, the single-stranded oligonucleotides used as transmission signals within circuits are highly susceptible to interference from the myriad natural biomolecules and degradation by nucleases, making it challenging for three-way strand displacement–based circuits to function reliably in cells, thus limiting their intracellular applications to relatively simple circuits (*33*–*39*).

As an alternative approach, Seelig and colleagues (*40*) have shown that toehold-mediated four-way strand exchange reactions can be adapted to mammalian cells, enabling AND and OR logic gates to directly interact with endogenous mRNAs. This approach leverages the predominately double-stranded nature of four-way strand exchange to minimize cross-talk with other nucleic acids in complex cellular environments, offering the unique advantages of strand exchange of nucleic acids to construct intracellular control circuitry (*40*–*46*). However, these four-way strand exchange-based gates were designed to be simple and exhibit low integrability, and challenging gate structure design was required to extend to other complex algorithm function, hindering the further scaling of nucleic acid circuits to the complexity required for advanced computations in living cells.

**Fig. 1.**
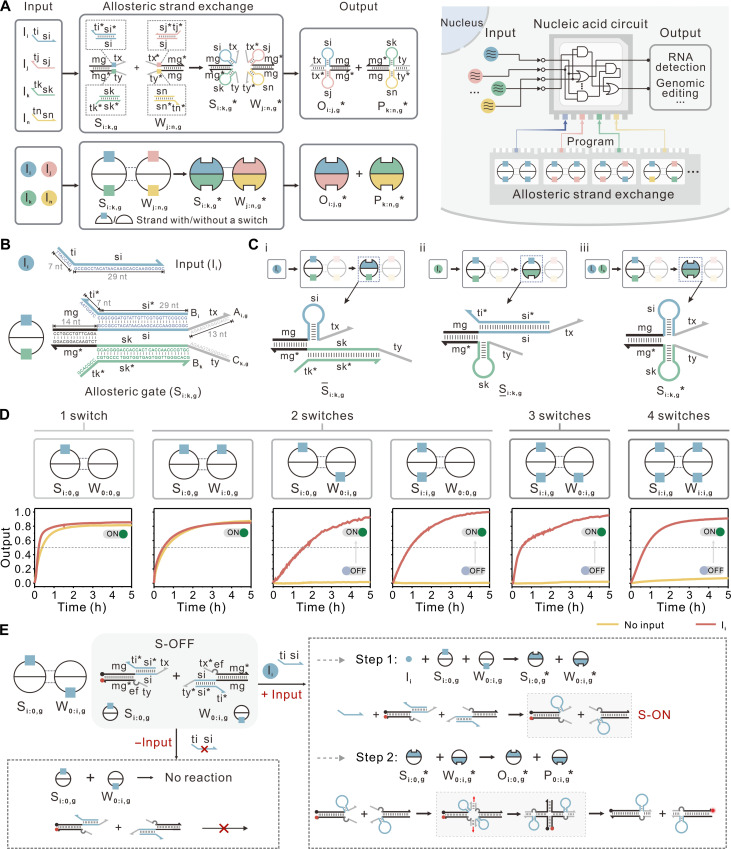
ASE reaction mechanism. (**A**) Schematic of ASE reactions. Top: DNA implementation of the ASE. Colored lines indicate DNA strands at the domain level, with arrows marking their 3′ ends. Each elementary functional domain is denoted by symbols without subscript, with asterisks indicating a complementary sequence. Symbols with subscripts indicate distinct DNA species, with asterisks in the names indicating that all molecular switches within gates are activated. When no molecular switch is inserted at the corresponding position, the corresponding subscripts i, j, k, or n is set to 0. Bottom: An abstract schematic diagram. The squares indicate programmable allosteric modules, with colors indicating distinct molecular switches. Right column: ASE serves as basic primitive to program complex intracellular circuits that perform Boolean logic to accomplish tasks. (**B** and **C**) Molecular details of the ASE system. The underscore or overline in the symbols indicates the specific position of the activated molecular switch (top or bottom strand) when the gate is in a partially activated state. (**D**) Characterization of the ASE. One of the gates is functionalized with a fluorophore/quencher pair. On completion of the toehold-mediated four-way branch migration, the fluorophore is delocalized from the quencher and generates an increased fluorescence signal. The dashed gray line marks the threshold value of 0.5. Signals above threshold were identified as ON; otherwise, they were identified as OFF. h, hours. (**E**) Mechanism proposed for the ASE. The dark gray line indicates a 2–nucleotide (nt) spacer inserted between toehold and recognition domains. Arrows indicate the flows of the reactions. Red and black dots denote fluorophore and quencher, respectively. More details are shown in figs. S2 to S4. The initial concentration of S_i:i,g_ and W_i:i,g_ is 1×; the initial concentration of input strand I_i_ is either 0× or 5× (standard concentration 1× = 50 nM).

Here, we present a mechanism, the allosteric strand exchange (ASE) as a generalization of four-way strand exchange, enabling to scale up nucleic acid computation in mammalian cells (Fig. 1A). We first systematically investigate the effect of the conformational variations and interactions of ASE and establish the design rules to tune its reaction kinetics, allowing the effective de novo design of circuits in a modular manner. Using ASE as a molecular primitive, we demonstrate a scalable nucleic acid circuit architecture that can implement complex Boolean logic functions within a single circuit layer, including AND, OR, AND-OR, and a four-input and an eight-input logic expression. Moreover, we demonstrate that a two-input ASE-based intracellular circuit can perform AND logic to specifically detect mRNAs in mammalian cells. We then construct an ASE-based intracellular multi-input molecular classifier that simultaneously senses the levels of four endogenous mRNA species to monitor cell reprogramming processes. We further show that ASE-based intracellular circuits can be easily reprogrammed to integrate with CRISPR-Cas9 systems for controllable gene editing, paving the way for efficient and tailored fabrication of intracellular biocomputing devices for biological applications.

## RESULTS

### ASE reactions

The principle of the basic ASE reaction is depicted in Fig. 1A, in which conformational transitions can be used for programmable regulation of strand exchange kinetics. The ASE system consists of allosteric gates (S_i:k,g_ and W_j:n,g_) that are two primarily double-stranded nucleic acid molecules and single-stranded inputs I_i_ (Fig. 1B and fig. S1). The predominantly double-stranded configuration of the gate structure design enables to minimize undesired cross-talk with other nucleic acids in complex cellular environments (*42*, *43*). In this design, molecular switches (dashed boxes in Fig. 1A), serving as allosteric modules, can be selectively inserted between the toehold and recognition domains to form the allosteric gates, in which a toehold on these switches allows the input to bind and activate switches (Fig. 1C), allowing initiation of a spontaneous intramolecular conformational change to form the hairpin loop structure and leading to the proximity of the toehold and recognition domains. As a result, the reaction then proceeds through a toehold-mediated four-way branch migration, which results in the exchange of strands between gates.

To systematically investigate the reaction mechanism of ASE, we designed and tested six different sets of allosteric gates in cell-free settings, each varying in the position and number of inserted molecular switches while maintaining the same switch sequence (Fig. 1D and figs. S2 to S4). Comparisons of reaction kinetics of ASE in all cases led to the several observations. First, when only one or two molecular switches were inserted into one or both gates to force only one toehold and recognition domain apart (figs. S2 and S3A), the similar kinetic behaviors were observed regardless of the presence of the input, as shown in the first two plots of Fig. 1D. This indicates that the binding of one toehold is sufficient to initiate a four-way branch migration between the two gates (figs. S2 and S3A), which is consistent with the observations in previous work (*41*). Second, when multiple molecular switches were inserted at specific positions in two gates to force both toehold and recognition domains apart (figs. S3B and S4), a strong fluorescence increase was observed only in the presence of the input and the reaction kinetics increased with the number of switches, as shown in the last four plots of Fig. 1D. This may be due to a substantial asymmetry between the two gates, resulting in increased steric hindrance from the hairpin loop, which makes strand exchange energetically less favorable. In the absence of the input, the molecular switches maintain a rigid, linear double-helix structure, rendering the reaction thermodynamically unfavorable. In the presence of the input, the dominant reaction path of ASE is sequential, where input binding to gates induces a conformational change, enabling both toeholds to hybridize and initiate four-way branch migration, ultimately leading to strand exchange between the two gates (Fig. 1E and figs. S3B and S4). We further investigated the influence of spacer length of gates on reaction kinetics and observed that optimizing internal diffusion processes effectively lowers the energy barrier, thereby enhancing the reaction rate (fig. S5). Together, these results demonstrate that the allosteric modules in each gate can be independently programmed to respond to distinct inputs and cooperatively control the kinetics of ASE reaction through conformational change, which exhibits additional design modularity, flexibility, and orthogonality.

We further demonstrate that the ASE reactions can work reliably in mammalian cells. The delivery regime was first established, where lipid-based transfection reagent Lipofectamine 3000 (L3K) was selected to stably package gates and input strand independently. We found that L3K could effectively prevent the interaction between the prepackaged components before entering the cells (fig. S6). Since the efficiency of ASE activation could be greatly limited by heterogeneities in the subcellular distributions of gates and input strands, we assessed their colocalization efficiency in cells using cotransfection and sequential transfection methods. Both components carried active fluorophores [Alexa Fluor 488 (AF488) or cyanine 5 (Cy5)] were delivered to human embryonic kidney (HEK) 293T cells using both transfection methods to track their intracellular concentrations. As a control, we transfected premixed components. Colocalization efficiency was notably affected by the transfection method, decreasing from ∼62% in cotransfection to ∼35% in sequential transfection (fig. S7, A and B). We then compared the efficiency of ASE activation using DNA components with that of 2′-*O*-methyl (2′OMe) RNA components in cells. The experimental data show that replacing DNA with 2′OMe RNA improved ASE activation by ∼6.5-fold and reduced false positives, while maintaining cell viability (figs. S7, C and D, and S8). This improvement is likely due to the enhanced stability of 2′OMe RNA against nuclease degradation, making it better suited for live cell studies (*47*–*49*). A time-course experiment indicated continued activation of the 2′OMe RNA components up to 6 hours (fig. S9).

### Implementation of complex logic computations with ASE

**Fig. 2.**
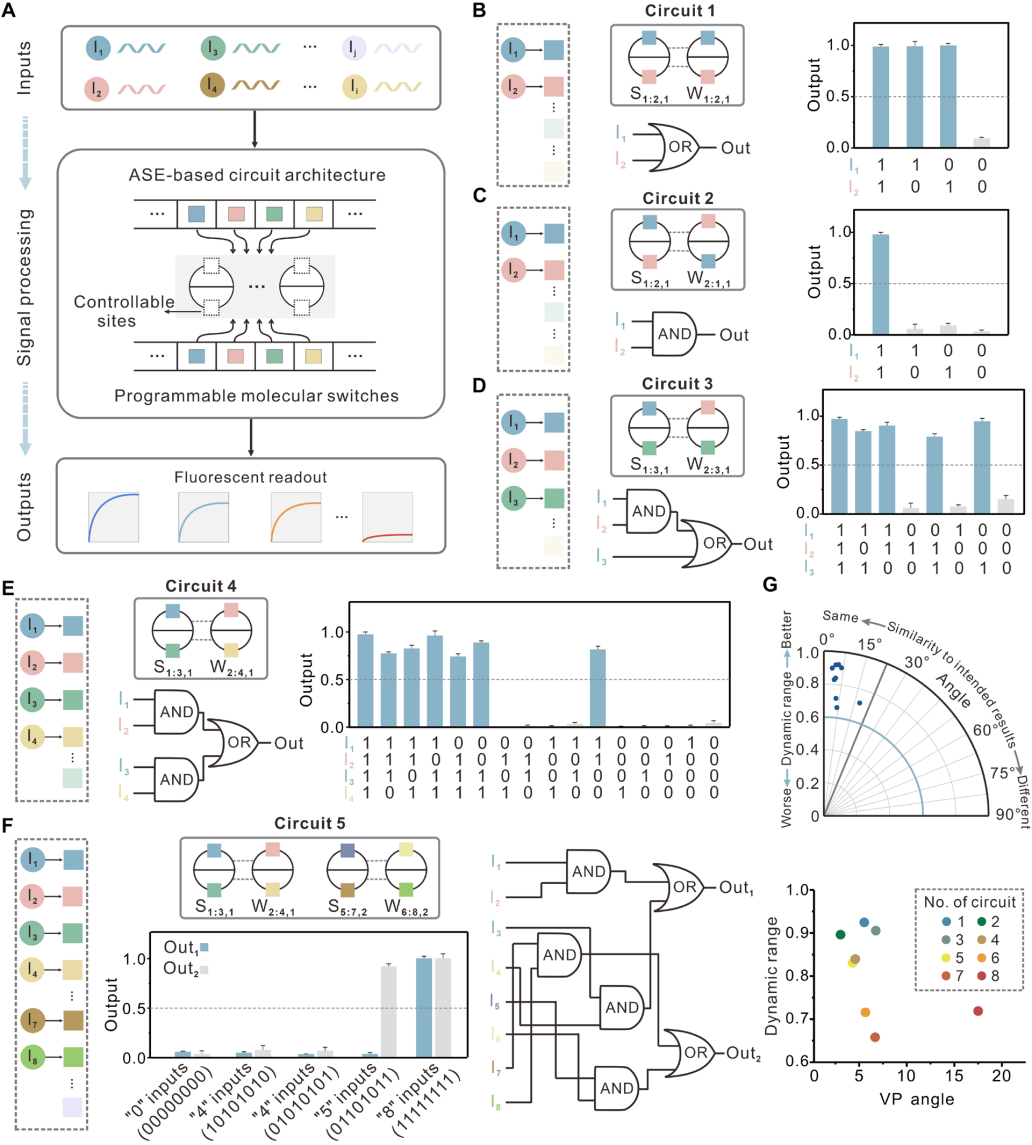
Designing the ASE-based circuit for complex logic computation. (**A**) Illustration depicting the programming of the ASE-based circuit with orthogonal molecular switch design. There are two controllable sites (dashed boxes) for each basic component of circuit. Different colored squares refer to molecular switches with distinct DNA sequences. (**B** and **C**) Two-input elementary logic computation. (**D**) Evaluation of a three-input ASE-based circuit that computes AND-OR logic function. (**E**) Evaluation of a four-input ASE-based circuit that features two ANDs coupled to one OR gate. Left column: Schematic representation of ASE-based circuit and corresponding logic gate diagram. Right column: The characterization of circuits for different input combinations after 5 hours (with blue and gray histograms denoting ON and OFF signals, respectively). (**F**) An eight-input ASE-based circuit. The blue and gray histograms correspond to the outputs of Out_1_ and Out_2_, respectively. Fluorescein amidite (FAM)/1-Dimethoxytrityloxy-3-{O-[N-4’-carboxy-4-(dimethylamino-azobenzene)-3-aminopropyl]}-propyl-2-O-succinoyl-long chain alkylamino (Dabcyl) and Cy5/black hole quencher 3 (BHQ3) fluorophore/quencher pairs were used for the characterization of the circuit. The dashed gray line marks the threshold value of 0.5. Error bars indicate SDs (means ± SD, *n* = 3). The DNA implementation of corresponding ASE-based circuit and its detailed reaction pathways are shown in figs. S10 to S14. (**G**) Top: VP angles between each signal vector and corresponding expected truth table vectors are plotted against the corresponding dynamic range values; *n* = 8. Bottom: Scatter plot of the corresponding dynamic ranges and VP angles. The initial concentration of S_i:k,g_ and W_j:n,g_ is 1×; the initial concentration of input strand I_i_ is either 0× (0, logic OFF) or 5× (1, logic ON). The standard concentration is 1× = 50 nM.

Next, we demonstrate that the ASE can be adopted as a basic primitive for engineering the scalable nucleic acid circuit architecture, enabling the complex multi-input logic processing in mammalian cells. This ASE-based circuit can be implemented by parallelly integrating multiple ASE reactions, in which the allosteric gate motifs can be used as the unified, modular components. This circuit architecture allows the flexible programming of multiple orthogonal molecular switches to diversify reaction pathways, which further expanding the circuit’s functionality within a single layer (Fig. 2A). For an ASE-based circuit that performs the *N* input–*M* output logic computation, *N* molecular switches with orthogonal sequences are designed to respond to *N* inputs, while *M* allosteric gates with distinct fluorophore/quencher pairs are used to read the output signals. Crucially, these molecular switches are carefully distributed at different positions among multiple allosteric gates, allowing the input-triggered conformational changes to control the kinetics of parallel ASE reactions and thereby enabling the desired logic functions at the molecular level.

For experimental demonstrations, we designed and tested a series of ASE-based circuits that enable various Boolean logic functions to be carried out in a single-layer circuit topology (Fig. 2, B to F, and figs. S10 to S14). We started the experimental demonstration with the implementation of two-input logic function. The circuit topologies, shown in Fig. 2 (B and C), each consist of two allosteric gates and are driven by a single ASE reaction (fig. S10). Two distinct molecular switches, designed to respond to two inputs, are inserted at specific positions to build the circuits that compute the logic function OR or AND. As the circuit runs, specific input combinations simultaneously trigger the conformational changes, enabling the initiation of four-way branch migration through the binding of one or both toeholds and resulting in the signal exchange. In addition, we showed that our architecture supports multiple circuit design configurations to realize the same computational function, introducing extra design flexibility (fig. S11). By simply increasing the number of orthogonal molecular switches, multi-input logic functions can be implemented while maintaining the same circuit scale, offering a facile way to scale the circuit functionality. A two-layer cascading AND-OR (Fig. 2D and figs. S12 and S13) and a four-input logic expression (Fig. 2E and fig. S14) were demonstrated. Furthermore, we integrated two parallel ASE reactions into a network and implemented an ASE-based circuit capable of computing a complex eight-input logic expression, equivalent to a moderate-scale cascaded circuit with six gates, further demonstrating the scalability of our architecture (Fig. 2F). We quantified the robustness of all circuits using vector proximity (VP) and dynamic range, and all data showed the reliable computation of ASE-based circuits (Fig. 2G). Overall, this architecture allows flexibly programmable conformational changes to parallelly control signal pathways of reaction network, enabling the implementation of complex logic computations in a compact, single-layer circuit.

### ASE-based intracellular AND logic for mRNA detection

**Fig. 3.**
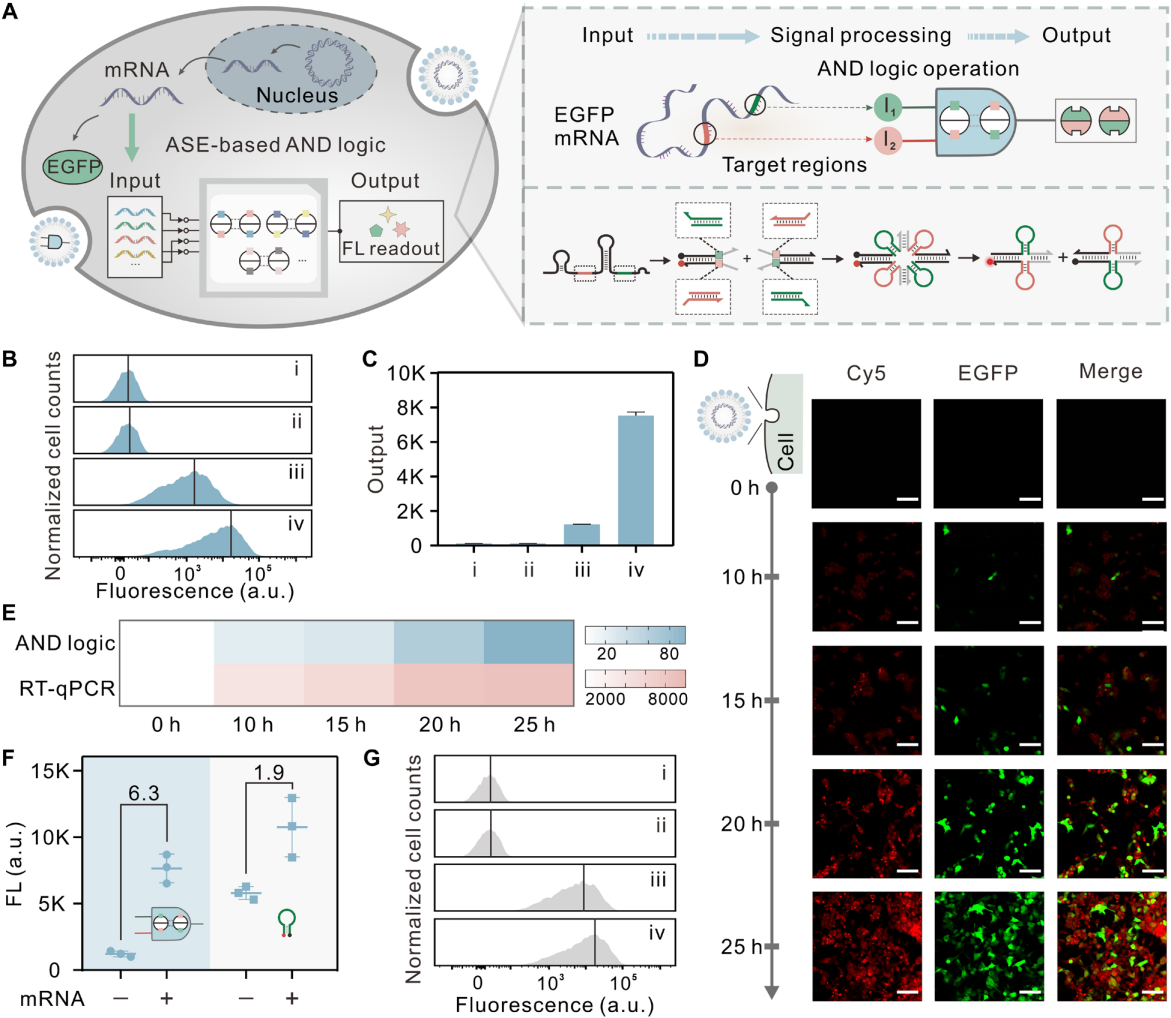
ASE-based intracellular AND logic for mRNA detection in mammalian cells. (**A**) Schematic of the intracellular AND logic for mRNA detection. Two molecular switches are programmed to response to two separate sites (indicated by colored lines) on the mRNA. pEGFP-N1 plasmids were transfected into HEK293T cells to stably transcribe EGFP mRNA as input. FL, fluorescence. (**B** and **C**) Flow cytometry measurements of two-input ASE-based circuit. Each histogram shows the cell counts versus fluorescence (the black line indicates the mean value) (B). The histograms correspond to cells treated with nothing (i), pEGFP-N1 plasmids alone (ii), circuit alone (iii), and circuit + pEGFP-N1 plasmids (iv). Bar graphs were obtained from the mean fluorescence of cells measured via flow cytometry (C). Error bars indicate SDs (means ± SD, *n* = 3). a.u., arbitrary units. (**D**) Confocal microscopy imaging of HEK293T cells transfected with pEGFP-N1 plasmids and circuit. A Cy5/BHQ3 pair was used for the characterization of circuit in cells. The timeline is shown on the left, indicating the time after transfection of the plasmid. The circuit components were cotransfected into the cells for the last 6 hours in each case. Scale bars, 100 μm. (**E**) The relative Cy5 fluorescence intensity and relative expression levels obtained from flow cytometry and RT-qPCR. Same experimental condition settings were kept as (D) for all time points. Color scales for heatmap indicate relative Cy5 fluorescence intensity and relative gene expression levels. (**F**) Plots of Cy5 fluorescence intensity obtained from flow cytometry, when EGFP mRNA detected using ASE-based circuit and conventional molecular beacon (MB). Error bars indicate SDs (means ± SD, *n* = 3). (**G**) Flow cytometry measurements of MB in cells. Each histogram shows the cell counts versus fluorescence (the black line indicates the mean value). The histograms correspond to cells treated with nothing (i), pEGFP-N1 plasmids alone (ii), MB alone (iii), and MB + pEGFP-N1 plasmids (iv).

We next demonstrate that the ASE-based circuits can be used to specifically detect mRNAs in mammalian cells through AND logic (Fig. 3A). To design an intracellular circuit targeting specific mRNA species in cells, we select two distinct target regions of each mRNA as the inputs to facilitate the specific target recognition and effective circuit activation. Two key issues must be considered when selecting these two target regions from each mRNA. First, unique sequences for each mRNA are identified using basic local alignment search tool analysis (*50*) to minimize off-target binding. Second, to ensure the circuit accessibility to target sites, the target regions that lie outside of complex secondary structures in mRNAs are selected. Our circuit architecture features unique orthogonal design in allosteric modules that can be designed to accept trigger mRNAs with arbitrary sequences, enabling them, in principle, to recognize different mRNAs simply altering the sequence of the allosteric modules.

As shown in Fig. 3A, the logical detection of enhanced green fluorescent protein (EGFP) mRNA was successfully demonstrated using the two-input ASE-based circuit in HEK293T cells. This circuit computes AND logic function, with two specific target regions of EGFP mRNA selected as the inputs for the circuit. This intracellular circuit can be triggered only upon simultaneously hybridizing to both target regions of mRNA, enabling simultaneous monitoring of circuit output via Cy5 and transcription of the target species through EGFP fluorescence. Figure 3 (B and C) shows the Cy5 fluorescence levels in the presence or absence of the trigger mRNA, with control measurements for comparison. The flow cytometric quantification revealed a ∼6.3-fold activation of the circuit only in the presence of target mRNA, indicating the efficacy of circuit in detecting mRNAs in living cells. We also evaluated the performance of this circuit in detecting mRNA at varying expression levels by measuring the targets at different time points after transfection, as mRNA expression levels varied over time. A steady increase in circuit activation level was observed as incubation time increase to 25 hours, which correlated well with the increase in mRNA expression levels measured by reverse transcription quantitative polymerase chain reaction (RT-qPCR) (Fig. 3, D and E, and fig. S15). These results demonstrate that our strategy of mRNA detection is specific, enabling the circuit to perform logical analysis of relative changes in mRNA expression. In addition, we compared this strategy with the conventional molecular beacon (MB) strategy and found that the ON/OFF ratio of our circuit is ∼4.4-fold higher than that of the MB probe, indicating its superior performance of low background binding and high specificity in mRNA detection in living cells (Fig. 3, F and G).

### ASE-based intracellular multi-input molecular classifier for cell type identification

**Fig. 4.**
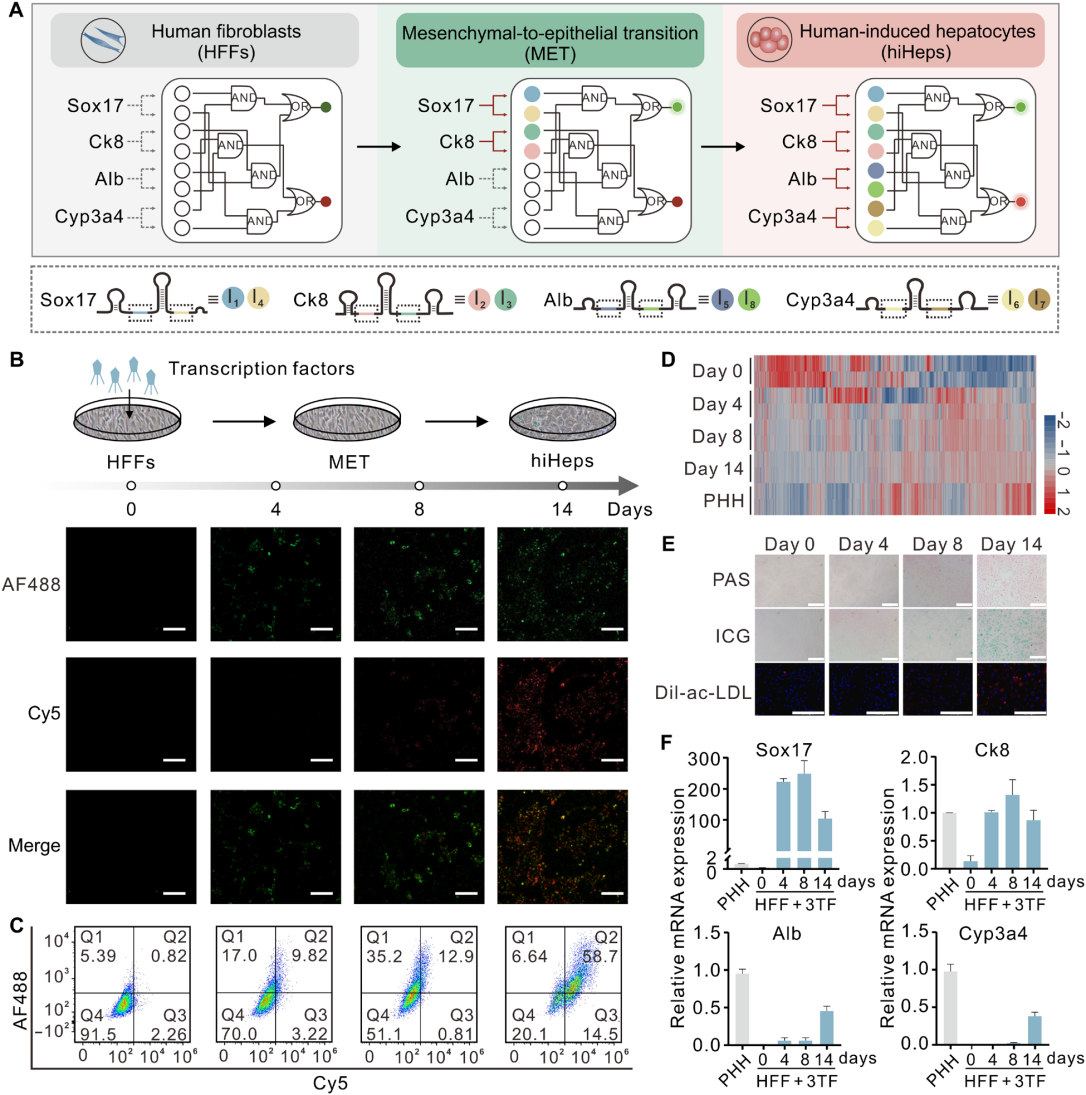
Engineering multi-input molecular classifier to monitor cellular reprogramming. (**A**) Schematic illustration of intracellular logic computation for in situ monitoring of dynamic cell reprogramming events. Changes in mRNA expression pattern along the reprogramming procedure can be used to track the reprogramming process of HFFs. Two separate sites (indicated by colored lines) on each endogenous mRNA are used as inputs for the classifier circuit. (**B**) Top: Schematic diagram depicting the experimental design for the culture and reprogramming of HFFs. The direct reprogramming of HFFs to hiHeps was done by three transcription factors (3TF) (i.e., FOXA3, HNF1A, and HNF4A). The culture medium was replaced with hepatocyte maintenance medium (HMM) 4 days after infection. Reprogrammed cells were characterized 0 to 14 days after induction. Bottom: Representative confocal microscopy images showing the behavior of the circuit along the reprogramming procedure. All circuit components were cotransfected into the cells at distinct time points. Two fluorophore/quencher pairs were used for the characterization of the circuit in cells. Scale bars, 100 μm. (**C**) Flow cytometry results. (**D**) Gene expression profile analysis of reprogrammed cells during hepatic conversion as measured by RNA sequencing. Primary human hepatocytes (PHHs) cultured for 48 hours were used as positive control. The color scale represents the log-normalized expression levels of each gene across cells. (**E**) Functional test including periodic acid–Schiff (PAS) staining (magenta; top), indocyanine green (ICG) uptake assay (green; middle), and intake of acetylated low-density lipoprotein labeled with a fluorescent probe, 1,1′-dioctadecyl-3,3,3′,3′-tetramethylindocarbocyanine perchlorate (Dil-ac-LDL) (red; bottom). Scale bars, 200 μm. (**F**) The analysis of relative expression levels for four endogenous mRNAs during hepatic conversion as measured by RT-qPCR. PHHs cultured for 48 hours were used as positive controls. Error bars indicate SDs (means ± SD, *n* = 3).

We further demonstrate the construction of the multi-input molecular classifier based on ASE, capable of recognizing and responding to multiple endogenous mRNAs for efficient multi-input information processing in mammalian cells. Specifically, we designed an eight-input classifier that simultaneously senses four endogenous mRNA species to discriminate cell types, all without the need for cell dissociation or the risk of mutagenesis (Fig. 4A). We evaluated the circuit’s performance in monitoring the direct reprogramming process of human fibroblasts (HFFs) into desired cell type, focusing on key events including mesenchymal-epithelial transition (MET) and transdifferentiation (*51*, *52*). Four pivotal biomarkers (i.e., Ck8, Sox17, Alb, and Cyp3a4 mRNA) related with these two events were chosen as inputs for the intracellular classifier circuit (*51*, *53*, *54*), enabling complex logical computations to be performed on a set of mRNAs to identify cell states in situ within live cells. As shown in Fig. 4 (B and C), as cells underwent reprogramming, the circuit was progressively activated in response to the increased mRNA expression levels, leading to a gradual enhancement of two fluorescence signals (AF488 and Cy5). We first observed an obvious AF488 fluorescence signal at day 4, which was still present at day 8, indicating the activation of MET process during the cell reprogramming. Subsequently, at day 14, an enhanced Cy5 fluorescence signal was observed, suggesting the successful transdifferentiation of HFFs into human-induced hepatocytes (hiHeps). Meanwhile, the negligible Cy5 fluorescent signal was observed in HFFs that did not undergo reprogramming (fig. S16). Figure 4 (D and E) shows the global expression profile analysis and in vitro functional analyses along the cell reprogramming period, confirming the successful direct reprogramming of HFFs into hiHeps that have hepatic gene expression pattern and mature functions (fig. S17). Furthermore, we found that the circuit activation level matched the trend of mRNA expression as measured by RT-qPCR (Fig. 4F), which further demonstrates the ability of our classifier circuit to efficiently monitor dynamic cellular processes by performing logical analysis of dynamic expression of multiple endogenous mRNAs.

### Reprogramming ASE-based intracellular circuit to control CRISPR-Cas9

**Fig. 5.**
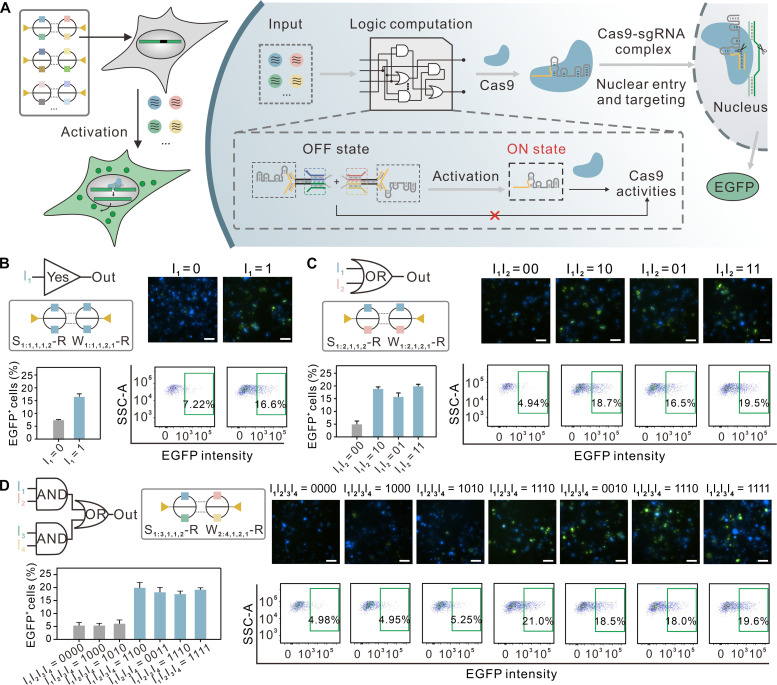
Reprogramming ASE-based intracellular circuit for control of Cas9 activity in living cells. (**A**) Design schematic of the ASE-based intracellular circuit that controls the targeting activity of Cas9 for genetic editing. The work principle of circuit is shown on the right column. One 18-nt region (indicated by the yellow lines) of sgRNA was chosen for conformation engineering to construct multi-input activation circuits. (**B** to **D**) The multi-input ASE-based circuits for sgRNA activation. HEK293T cells were first transfected with Cas9 (pX330-U6-Chimeric_BB-CBh-hSpCas9 vector) and a deactivated EGFP gene disrupted by the insertion of a stop codon and a target genomic sequence (pCMV-BFP-P2A-EGFP-SSA vector). Twenty-four hours later, the cells were cotransfected with the circuit components and inputs. For different input combinations, EGFP signal was assayed using confocal microscopy and flow cytometry after ∼72 hours. Scale bars, 100 μm. Error bars indicate SDs (means ± SD, *n* = 3).

The CRISPR-Cas9 system enables simple and efficient genome editing, providing a powerful tool for therapeutic applications in living organisms (*55*–*57*). However, achieving precise control over Cas9 gene-editing activity remains challenging. We demonstrate that an ASE-based intracellular circuit can interface with the CRISPR-Cas9 system by engineering the conformation of single guide RNA (sgRNA), enabling programmable control over Cas9 gene-editing activity (Fig. 5A). To achieve this, we reprogrammed each allosteric gate within circuits to incorporate a 9–nucleotide (nt) blocker region (highlighted by the yellow line in Fig. 5A and fig. S18), which binds to the self-hybridized region of the sgRNA to form sgRNA-gate complex, inducing a conformational change that switches the sgRNA to an OFF state and inhibits its target recognizing activity (fig. S18). The originally bound sgRNA can be released upon the arrival of corresponding input strands and then restored its ON state activity. This design enables the independently programming of multiple orthogonal molecular switches to execute complex multi-input logic operations on sgRNA, thereby regulating Cas9 activity in response to signals of interest. By only reprogramming the number of orthogonal molecular switches, we demonstrated three distinct ASE-based circuits on a sgRNA molecule, capable of performing YES, OR, and four-input logic operations computation (Fig. 5, B to D, and figs. S19 to S21). We experimentally tested the ability of these circuits to logically control Cas9 activation by incubating them with HEK293T cells cotransfected with two plasmids: one encoding Cas9 and the other encoding a deactivated EGFP where the EGFP-coding sequence was disrupted by the insertion of a stop codon and a target genomic sequence. The sgRNA was designed to hybridize with the target genomic sequence within the EGFP gene, directing Cas9 to cleave the target region and thereby restoring EGFP gene expression (Fig. 5A and fig. S18). As shown in Fig. 5 (B to D) and fig. S22, we observed a ~17 to 21% increase in fluorescent cells (EGFP positive) upon circuit activation, while substantially lower fluorescence was detected when the circuit was inactivated. In addition, we found that scaling the circuit to multi-input logic expression had a negligible effect on the target binding efficiency, as our circuit architecture allows for the functionality expansion within a single layer. These results collectively suggest that our ASE-based circuit architecture offer a simple and efficient strategy for conditional activation of CRISPR-based systems in living cells.

## DISCUSSION

In this work, we demonstrated a mechanism that enables scaling up the complexity of nucleic acid circuitry in mammalian cells in a modular manner. The ASE we designed leverages independent conformational transitions to cooperatively regulate strand exchange kinetics, which offers additional design modularity, flexibility, orthogonality, and low cross-talk with unrelated endogenous information. On the basis of this mechanism, we developed a scalable ASE-based circuit architecture that can reliably compute complex multi-input logic functions in living cells. This circuit architecture is enabled by the parallel integration of multiple ASE reactions incorporated with flexible programming of allosteric modules, allowing the implementation of combinatorial Boolean logic within a single circuit layer. This feature enables more complex circuit topologies to be encoded with fewer components, making the architecture well suited for scaling up to larger and more sophisticated intracellular circuits. Furthermore, we demonstrated that these circuits can directly interact with cellular mRNA and further showed its ability to logically analyze the levels of multiple distinct mRNA species, highlighting their potential to simultaneously integrate endogenous cellular information and enable efficient signal processing in living cells for synthetic biology applications. The modular design of allosteric gates allows for the customization of allosteric modules to match the target sequences, facilitating their adaptation for in vivo detection of other endogenous nucleic acids and their integration with additional gates to develop complex intracellular nucleic acid circuits that can minimize off-target binding and enhance specificity against homologous sequences. Moreover, the ASE-based intracellular circuits can be easily reprogrammed to integrate with the CRISPR-Cas9 system for logic-based control of gene editing in cells, demonstrating the feasibility of engineering complex control devices capable of sensing a variety of native RNA sequences and, in response, regulating gene expression to precisely control cell function. We anticipate that the ASE-based circuit architecture presented here could provide an efficient strategy for constructing complex intracellular nucleic acid computing systems that can be tailored to respond to specific intracellular conditions and enable the programming of desired cellular behaviors, which might pave the way for advancing applications in diagnostics, therapeutics, and biotechnology.

## MATERIALS AND METHODS

### Materials

All oligonucleotides used in this study were provided by Sangon Biotech. All unmodified oligonucleotides were purified by ultra–polyacrylamide gel electrophoresis; and all oligonucleotides with fluorophore, quencher, or 2′OMe RNA modifications were purified by high-performance liquid chromatography and used without further purification. sgRNA was chemically synthesized by GenScript. All oligonucleotides were dissolved at 100 μM in tris-acetate-EDTA buffer containing 12.5 mM Mg^2+^ (1× TAE/Mg^2+^) and stored at 4°C for further use. L3K, TRIzol, and low-density lipoprotein from human plasma, acetylated, and 1,1′-dioctadecyl-3,3,3′,3′-tetramethylindocarbocyanine perchlorate complex (Dil-ac-LDL) were purchased from Invitrogen. Human albumin enzyme-linked immunosorbent assay (ELISA) quantitation kit was purchased from Bethyl Laboratory. Urea assay kit was purchased from QuantiChrom. Periodic acid–Schiff (PAS) staining system and indocyanine green (ICG) were purchased from Sigma-Aldrich. Moloney murine leukemia virus (MoMLV) reverse transcriptase; Cytochrome P450 1A2 (CYP1A2), CYP3A4, and CYP2C9 assay and screening systems were purchased from Promega. The pEGFP-N1 (B540189-0001) was purchased from Sangon Biotech. The psPAX2 (#12260), pWPI (#12254), pMD2.G (#12259), and pX330-U6-Chimeric_BB-CBh-hSpCas9 (#42230) plasmids were obtained from Addgene.

### Sequence design

Each allosteric gate has two long single strands (A_i,g_ and C_k,g_), which are complementary to the corresponding short single strands (B_i_) (fig. S1). Each long single strand consists of a 13-nt toehold (tx) domain and a 14-nt recognition (mg) domain, between which molecular switches can be selectively inserted. For each long single strands (A_i,g_ and C_k,g_), two nucleotides “TT” used as spacer are inserted between the toehold domains and recognition domains on demanded. Allosteric gates with distinct fluorophore/quencher pairs are used as reporters to read the output signals. To combine with CRISPR-Cas9 system, each allosteric gate was reprogrammed to incorporate a 9-nt blocker region within each long single strand (fig. S18A). All sequences were designed and examined using NUPACK (*58*) to check the secondary structures. The nucleotide sequences of all oligonucleotides used in this work are provided in tables S1 to S7.

### Assembly of the allosteric gates and sgRNA-gate complexes

The allosteric gates were assembled by mixing A_i,g_, C_k,g_, and B_i_ strands in 1× TAE/Mg^2+^ buffer to final concentrations of 5, 5, and 7.5 μM, respectively. Fluorescence strand and quencher strand were mixed at molar ratio of 1:1.2. This excess will ensure the formation of allosteric gates and imperfect stoichiometry, without changing the fluorescence baseline. The annealing processes were conducted in a thermal cycler (Life Technologies), starting with a 5-min heating up to 95°C, followed by gradual cooling to 20°C at a rate of 0.1°C per 8 s, and then kept at 4°C. To prepare sgRNA-gate complexes, the gates were separately synthesized. sgRNA molecules targeting EGFP were mixed and incubated with the annealed gates at a molar ratio of 1:1 at 45°C for 10 min, with an annealing procedure slowly from 45°C to room temperature at a rate of 0.1°C per 8 s.

### Plasmids construction

pCMV-BFP-P2A-EGFP-SSA vector was constructed through Gibson assembly (*59*). DNA polymerase KOD-Plus-Neo (Toyobo), MultiS One Step ClonExpress Kit (Vazyme), FastDigest Not I, and FastDigest Bsh TI (Thermo Fisher Scientific) were adopted.

### Cell culture and transfection

HEK293T, HEK293FT, and HFF cells were purchased from the Cell Bank of Type Culture Collection of the Chinese Academy of Sciences. PHHs were purchased from Lonza. HEK293T and HEK293FT cells were cultured in Dulbecco’s modified Eagle’s medium (DMEM) supplemented with 10% fetal bovine serum and penicillin-streptomycin (0.5 mg/ml). HFFs were maintained in HFF medium. PHHs were cultured in hepatocyte maintenance medium (HMM) (*51*). Cells were cultured in humidified atmosphere containing 5% CO_2_ at 37°C. L3K was used for transfecting allosteric gates and inputs. Allosteric gates were transfected at 1× (50 nM), and the inputs were transfected at 5× concentration. For cotransfection, allosteric gates and inputs were incubated with L3K independently and then simultaneously added to the cells. Transfection experiments were conducted in prewarmed Opti-MEM medium. Following 4 hours of transfection, the cells were washed twice with Dulbecco’s phosphate-buffered saline (DPBS). Subsequently, 500 μl of DMEM was added, and the cells were further incubated for an additional 2 hours. Sequential transfection was carried out at the same ratios of transfection reagent to allosteric gates and inputs in cotransfection. Transfection mixtures were prepared as above; after adding the first transfection mixture, the cells were incubated for 2 hours. Next, the cells were washed twice with DPBS, and the second transfection was carried out. Two hours later, the cells were washed twice with DPBS, and then DMEM was added. The cells were incubated for another 2 hours before the measurement (*40*). For genetic manipulation of EGFP in HEK293T cells, after overnight seeding and culturing, the cells were transfected with pX330-U6-Chimeric_BB-CBh-hSpCas9 and pCMV-BFP-P2A-EGFP-SSA vectors using L3K. After 24 hours of culture, sgRNA-gate complexes with or without corresponding inputs were cotransfected into the cells and incubated for 72 hours. The efficiency of gene editing was analyzed by confocal microscope and flow cytometry.

### Culture and direct reprogramming of human fetal fibroblasts

Lentivirus production and hiHep induction were performed as depicted previously by Huang *et al.* (*51*). Viruses were produced by introducing packaging plasmid psPAX2, modified pWPI plasmids carrying candidate genes forkhead box A3 (FOXA3), hepatocyte nuclear factor 1 homeobox A (HNF1A), and HNF4A), and enveloped plasmid pMD2.G into HEK293FT cells. HFFs were seeded on collagen-I–coated dishes and infected with lentiviruses.

### Cell-free kinetics assays

Fluorescence experiments were conducted using a spectrofluorometer (Fluorolog-max, HORIBA). The instrument allows four experiments to be run simultaneously. The temperature was kept at 25°C throughout the experiment. The excitation (Ex)/emission (Em) wavelengths for different fluorophores were set as follows: fluorescein amidite (FAM; 494 nm/518 nm), AF488 (490 nm/525 nm), and Cy5 (643 nm/667 nm). Before the experiment, all cuvettes were successively cleaned six times with distilled water, once with 70% ethanol, and then five times with distilled water. The concentrations of allosteric gates and inputs were 1× and 5×, respectively, with a standard 1× concentration of 50 nM. Parallel experiments for ASE reactions with varying numbers of inserted molecular switch were normalized together for data analysis. For each ASE-based circuit with different input combinations, all fluorescence values were normalized together for the readout of logic results, with the instrument-induced differences in fluorescence being negligible. For a given fluorophore, the maximum level (output = 1) was obtained from the highest signal produced from the reporter in the sets of parallel experiments. We used 0.5× as thresholds.

### Flow cytometry

HEK293T cells were plated at 50,000 cells per well in 24-well plates and cultured overnight. After transfection for predetermined time intervals, the culture medium was removed, and cells were washed twice with DPBS. Subsequently, cells were detached and resuspended in 0.5 ml of DPBS following trypsinization and washing. Last, the fluorescence intensities were detected using FACSCanto II cytometer (BD). Gates were drawn using side scatter area A (SSC-A) versus forward scatter area A (FSC-A) and kept the same throughout the experiments.

### Confocal microscopy

HEK293T cells were plated on glass coverslips 1 day before experiments. After staining with Hoechst 33242, the cells were washed twice with DPBS and imaged using a laser scanning confocal microscope (Leica TCS SP8). The wavelength sets were 633-nm Ex/ 660- to 730-nm Em for Cy5, 488-nm Ex/504- to 574-nm Em for AF488 and EGFP, and 405-nm Ex/425- to 498-nm Em for Hoechst 33242.

### Quantitative real-time PCR analysis

Total RNA was extracted from cells by TRIzol. For PHHs, mRNA was isolated from PHHs cultured for 24 hours after plating. cDNA conversion was performed with MoMLV reverse transcriptase. Quantitative real-time PCR was carried out to obtain *C*_T_ values of marker genes on CFX96 Touch Real-Time PCR Detection System (Bio-Rad). All data were performed with at least three repeats. Relative mRNA expression levels of marker genes were normalized to the expression level of housekeeping gene. Primer sequences are provided in table S7.

### Hepatic function assays

For analysis of albumin secretion, cells at days 0 to 14 and PHHs were incubated in hepatocyte culture medium (HCM) without bovine serum albumin for 24 hours. Supernatants were collected and stored at −80°C. Albumin secretion was measured using human albumin ELISA quantitation kit. Urea production was measured by urea assay kit. For PAS, Dil-ac-LDL, and ICG staining assays, PAS and Dil-ac-LDL were used following the manufacturer’s instructions. ICG uptake assay was performed by replacing the medium with ICG (1 mg/ml). After incubation at 37°C for 1 hour, cells were washed three times with DPBS. CYP activity assay was analyzed with P450-Glo CYP1A2, CYP3A4, and CYP2C9 assay kits (Promega) following the manufacturer’s instructions. For CYP metabolism assay, cells at days 8 and 14 were cultured in hiHep CYP450 induction medium (HIM) supplemented with rifampin (25 μM) for 48 hours. Total RNA was extracted from cells to measure the induction of CYP enzymes by RT-qPCR.

### RNA sequencing

Total RNA was extracted from cells by TRIzol. mRNA libraries were constructed using a deribosomalized, strand-specific strategy. Libraries were sequenced on the Illumina HiSeq Xten/NovaSeq platform, using a 2 × 150 paired-end sequencing strategy. Sequencing data have been deposited in the Sequence Read Archive under the accession number PRJNA844615. Trim_galore (version 0.6.4) was used to remove adapters from raw sequencing data and eliminate reads with a quality control of <25. Clean data were aligned to the human genome (GRCh38) using STAR (version STAR_2.6.1a). Gene expression and annotation were quantified using Stringtie (version 1.3.3) to the annotation file (Gencode.v35), providing the count of each transcript. For differential expression analysis, genes were normalized and differentially analyzed using DESeq2.

## Supplementary Materials

### This PDF file includes:

Figs. S1 to S22 Tables S1 to S8 References
