# Supplementary material for: Implementing complex nucleic acid circuits in living cells
Source: Sci Adv. 2025 Apr 30;11(18):eadv6512. doi: 10.1126/sciadv.adv6512 (PMC12042877; doi:10.1126/sciadv.adv6512)
Supplement: Supplementary file 1 — Figs. S1 to S22 Tables S1 to S8 References [file sciadv.adv6512_sm.pdf]

Science Advances
AAAS

Supplementary Materials for

Implementing complex nucleic acid circuits in living cells

Jiajia Sun *et al.*

Corresponding author: Hao Pei, peihao@chem.ecnu.edu.cn

Sci. Adv. **11**, eadv6512 (2025)
DOI: 10.1126/sciadv.adv6512

This PDF file includes:

Figs. S1 to S22
Tables S1 to S8
References

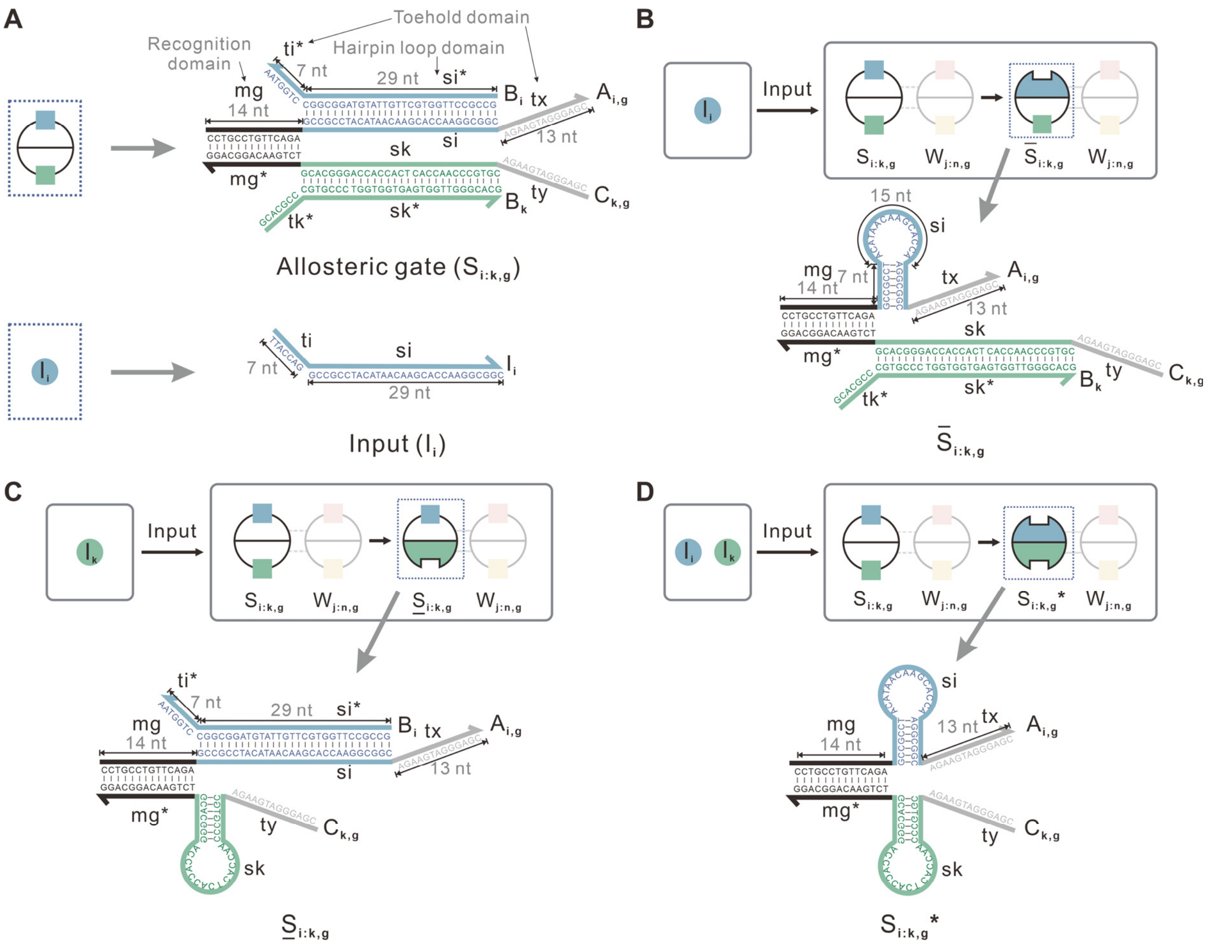

Fig. S1. Schematic illustration and molecular details of the ASE system. (**A**) Abstract diagram and molecular details for the ASE system. Our ASE system consists of two types of molecules: (1) allosteric gate (for example $S_{i:k,g}$) that has two long single strands ($A_{i,g}$ and $C_{k,g}$) complementary to the corresponding short single strands (B_i). Each long single strand consists of a 13-nt toehold domain (tx) and a 14-nt recognition domain (mg), between which molecular switches can be selectively inserted. Symbols with subscripts indicate distinct DNA species. In details, $S_{i:k,g}$ is an allosteric gate inserted with two molecular switches between the toehold and recognition domain (mg). (2) single-stranded input I_i. (**B-D**) Abstract diagram and molecular details for the activation of allosteric gate. For an allosteric gate $S_{i:k,g}$ with two distinct orthogonal molecular switches, the activation state of the gate varies depending on the combination of two inputs (I_i and I_k). When only I_i is present, the inserted molecular switch on the top strand is activated, and the allosteric gate is in a partially activated state, referred to here as $\bar{S}_{i:k,g}$ (**B**). When only I_k is present, the inserted molecular switch on the bottom strand is activated, and the allosteric gate is in another

partially activated state, referred to here as $\underline{S}_{i:k,g}$ (**C**). When both inputs (I_i and I_k) are present, both molecular switches are activated, and the allosteric gate is in the fully activated state, referred to here as $S_{i:k,g}$* (**D**). Thus, the underscore or overline in the symbols indicates the specific position of the activated molecular switch (top or bottom strand) when the gate is in a partially activated state, while symbols marked with an asterisk signify that all molecular switches within the DNA species are fully activated.

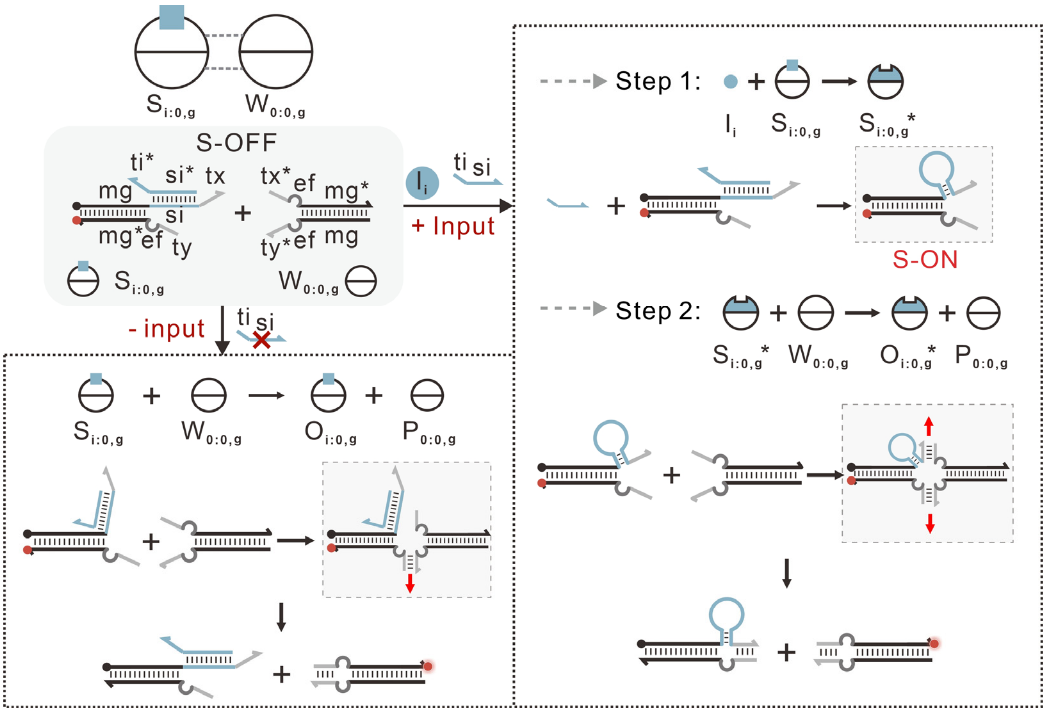

Fig. S2. Reaction pathways of ASE with one molecular switch inserted into one allosteric gate. One molecular switch was inserted into the allosteric gate to force one toehold (tx) and recognition domain (mg) apart. In the absence of the input (I_i), the binding of one toehold (ty) domain can initiate a four-way branch migration between two gates. In the presence of the input (I_i), we hypothesize that the ASE reaction may proceed along two distinct pathways, which could coexist within the reaction system. The binding of one toehold (ty) domain can initiate a four-way branch migration between two gates. Meanwhile, the input can bind and displace B_i to induce a conformational change of the molecular switch that brings the toehold and recognition domains into close proximity (step 1), enabling both toeholds to hybridize and initiate a four-way branch migration, ultimately leading to strand exchange between the two gates (step 2). Red and black dots denote fluorophore and quencher, respectively. Symbols with subscripts indicate distinct DNA species, with asterisks in the names indicating that all molecular switches within gates are activated. The dark gray line indicates a 2-nt spacer inserted between toehold and recognition domains. The black arrows indicate the flows of the reactions.

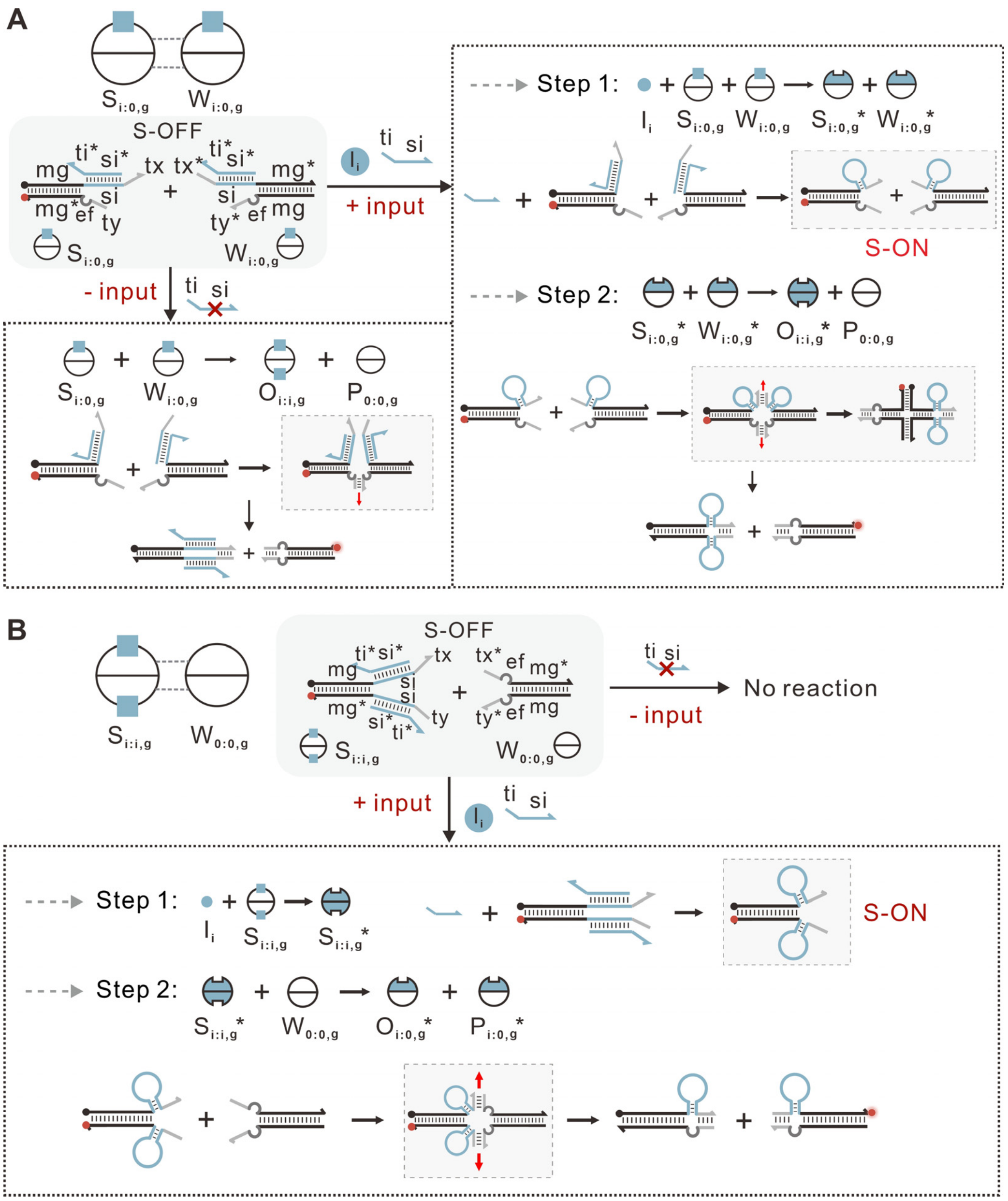

Fig. S3. Reaction pathways of ASE with two molecular switches inserted into allosteric gates. (**A**) Detailed schematics of ASE reaction pathways when two molecular switches are inserted into both allosteric gates. In the absence of the input (I_i), the binding of one toehold (ty) domain can initiate a four-way branch migration between the two gates. In the presence of the input (I_i), we hypothesize that the ASE reaction may proceed along two distinct pathways, which could coexist within the reaction system. The binding of one toehold (ty) domain can initiate a four-way branch migration between two gates. Meanwhile, the input can bind and displace B_i to induce a conformational change of two molecular switches that brings the toehold and recognition domains

into close proximity (Step 1), enabling both toeholds to hybridize and initiate a four-way branch migration, ultimately leading to strand exchange between the two gates (Step 2). (**B**) Scheme of ASE reaction pathways when two molecular switches are inserted into one allosteric gate. The rigid, linear double-helix structure of the two molecular switches in one allosteric gate forces the toehold and recognition domains apart. In the absence of input (I_i), the strand exchange is energetically less-favorable. In the presence of input (I_i), the reaction path of ASE includes two steps. First, the input binds and displaces B_i to induce a conformational change of two molecular switches, resulting in the formation of hairpin-loop structure to bring the toehold and recognition domains into close proximity (Step 1). Then, four-way branch migration is initiated by the binding of both toeholds to exchange strands. Red and black dots denote fluorophore and quencher, respectively. Symbols with subscripts indicate distinct DNA species, with asterisks in the names indicating that all molecular switches within gates are activated. The dark gray line indicates a 2-nt spacer inserted between toehold and recognition domains. The black arrows indicate the flows of the reactions.

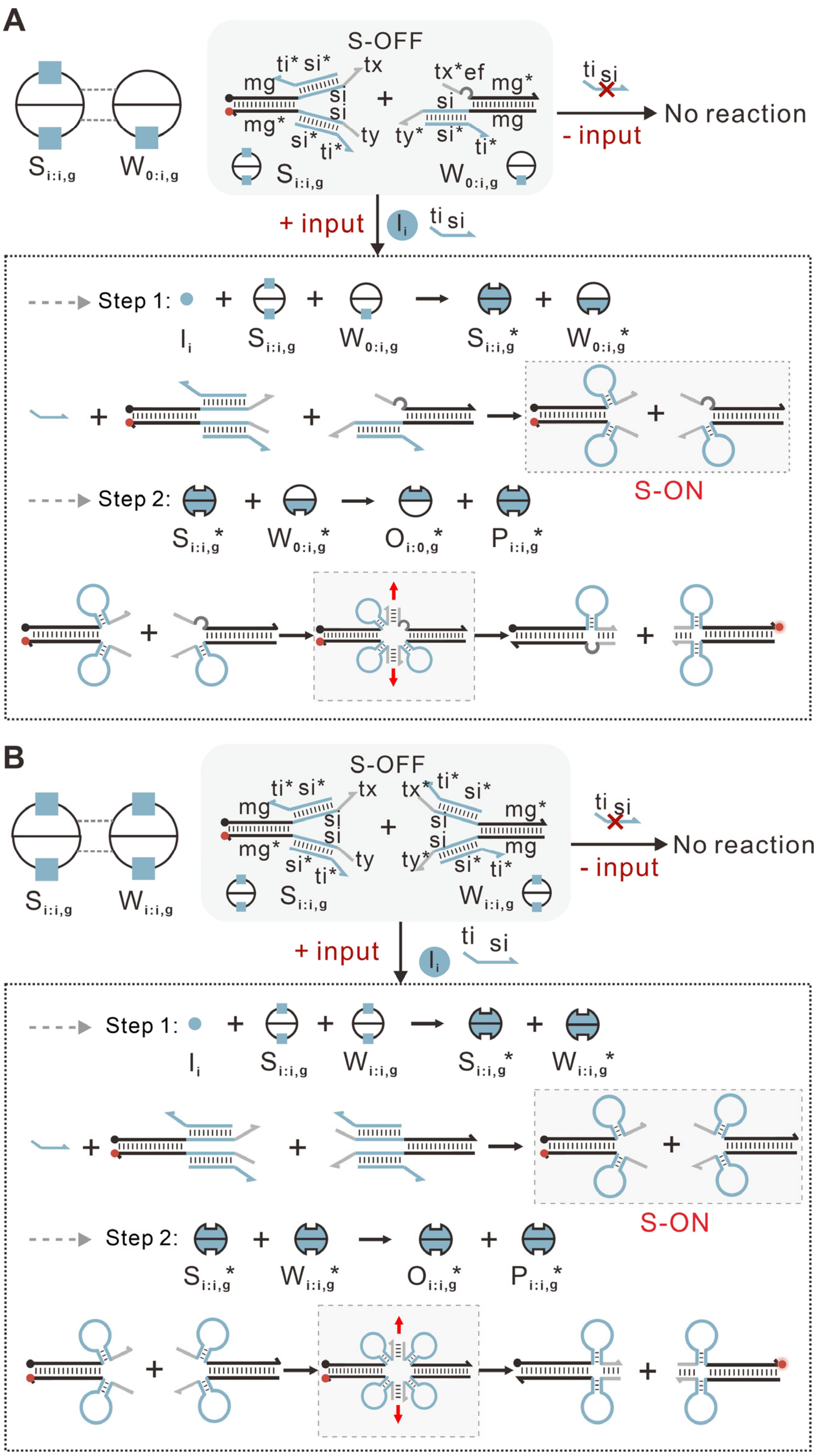

Fig. S4. Reaction pathways of ASE with three or four molecular switches inserted into allosteric gates. (**A-B**) The rigid, linear double-helix structure of the two molecular switches in

the allosteric gates forces the toehold and recognition domains apart, making the reaction thermodynamically unfavorable in the absence of input (I_i). In the presence of input (I_i), the reaction path of ASE includes two steps. First, the input binds and displaces B_i to induce a conformational change of three or four molecular switches, resulting in the formation of hairpin-loop structure to bring the toehold and recognition domains into close proximity (Step 1). Then, four-way branch migration is initiated by the binding of both toeholds to exchange strands. Red and black dots denote fluorophore and quencher, respectively. Symbols with subscripts indicate distinct DNA species, with asterisks in the names indicating that all molecular switches within gates are activated. The dark gray line indicates a 2-nt spacer inserted between toehold and recognition domains. The black arrows indicate the flows of the reactions.

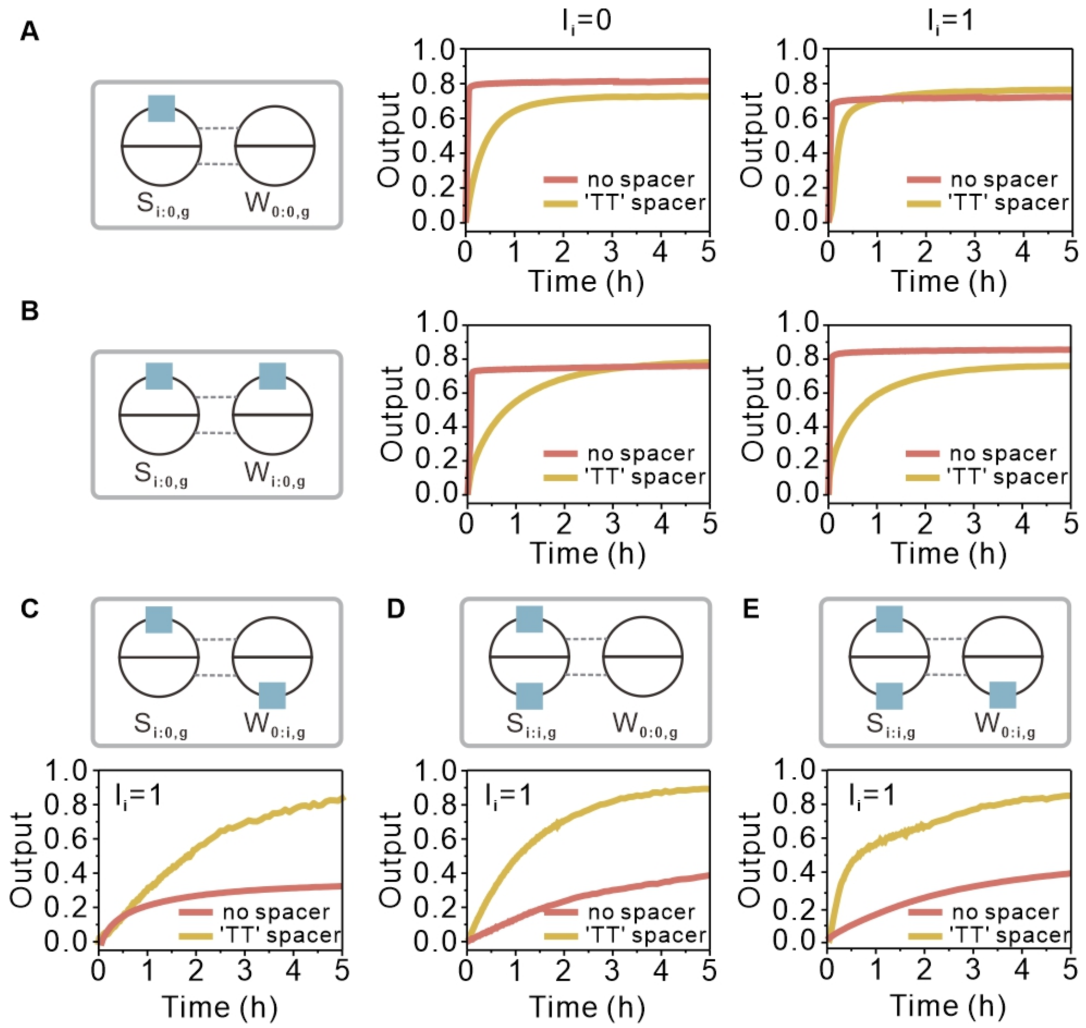

Fig. S5. The effect of spacer length on the ASE reaction kinetic. (A-E) Fluorescence kinetics data of ASE reaction with and without spacer. The length of spacer is either 0 or 2 nt. The ASE reactions were performed in 1× TAE/ Mg^{2+} buffer at 25 °C. The initial concentration of allosteric gates is 1×; the initial concentration of input strand I_i is either 0× or 5×. The standard concentration is 1× = 50 nM.

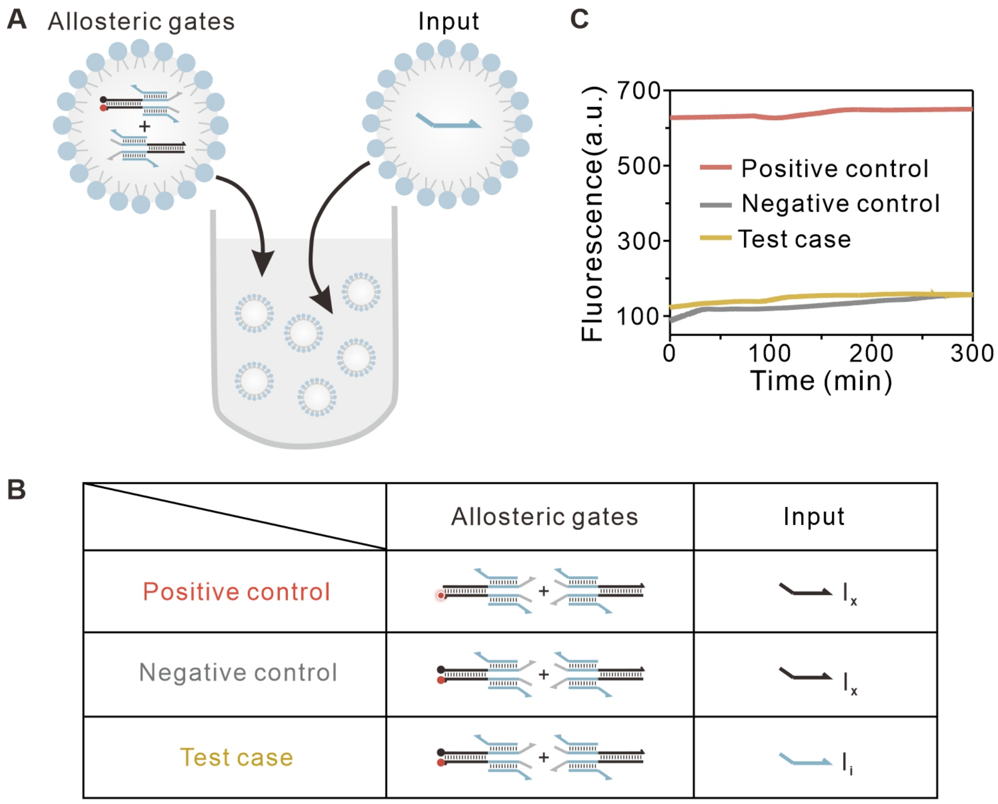

Fig. S6. *In vitro* interaction test between allosteric gates and input using L3K transfection reagent. (**A**) Scheme of *in vitro* mixing experiments. Allosteric gates and input strand were incubated separately with L3K for 15 minutes before mixing. (**B**) Detailed list of allosteric gate and input design. Positive control: one of the allosteric gates labeled with AF488 only, and the input was scrambled (I_x); Negative control: two allosteric gates were the same as the test case, and the input strand was scrambled (I_x). (**C**) *In vitro* kinetics of ASE. The experiments were conducted in Opti-MEM medium at 37 °C. Allosteric gates were at 1× and packaged using 2 μL L3K; input was at 5× and packaged using 4 μL L3K. The standard concentration is 1× = 50 nM.

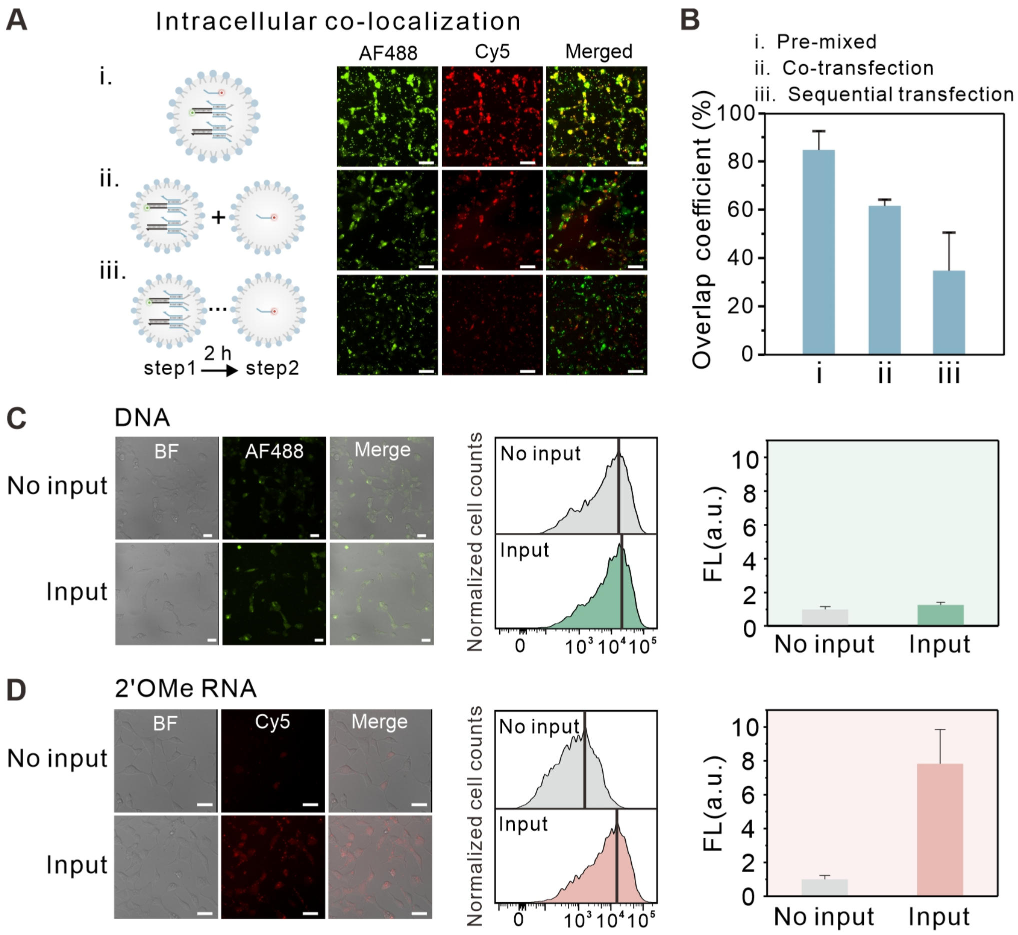

Fig. S7. Characterization of ASE in mammalian cells. (**A**) Optimization of delivery regime. Left: Delivery methods. i. Pre-mixed: two components respectively labelled with Cy5 (red) or AF488 (green) were mixed prior to incubation with L3K in the same tube; ii. Co-transfection: two components were incubated independently with L3K in different tubes and added to cells at the same time; iii. Sequential transfection: two components were incubated independently with L3K in different tubes while added to cells separately with a two-hour gap. Right: confocal images. The scale bars are 100 µm. (**B**) The overlap coefficient. Error bars show standard deviations calculated from at least two replicates. (**C-D**) Activation of ASE reactions under different chemical conditions. Left: representative confocal images. Middle: flow cytometry results. Right: mean fluorescence intensities in all conditions. The black lines indicate the mean fluorescence intensity for AF488 or Cy5, respectively. The fluorescence values of gates and input are normalized to inactive gates. Scale bars are 50 µm. Error bars indicate standard deviations (mean ± s.d., n = 3).

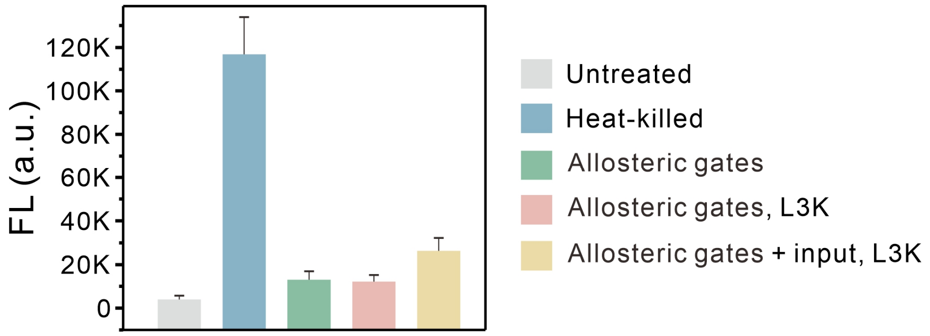

Fig. S8. Cell viability following transfection of ASE reaction. HEK293T cells were co-transfected with 2′OMe RNA components and input strand for 4 hours and then incubated in DMEM medium for an additional two hours. Heat-killed cells were incubated in 1× DPBS + 2% FBS at 65 °C. A fluorescent live-cell impermeant nucleic acid stain (Propidium Iodide) was used to measure the number of cells with damaged membranes following transfection. Cell viability was measured by flow cytometry. Error bars indicate standard deviations (mean ± s.d., n = 3).

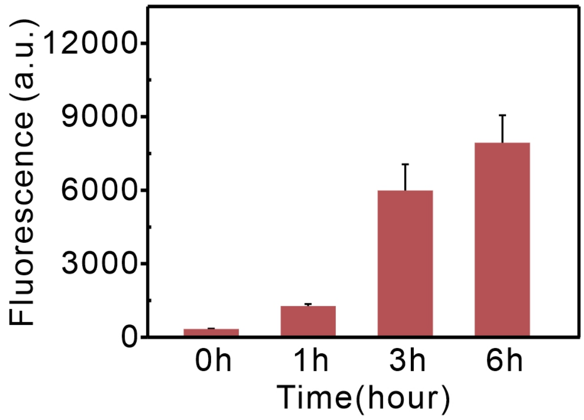

Fig. S9. Time-course of ASE activation in cells. HEK293T cells were co-transfected with 2′OMe RNA components and input strand as described and the cells were analyzed by flow cytometry at different time points post delivery (0 h, 1 h, 3 h and 6 h). Error bars indicate standard deviations (mean ± s.d., n = 3).

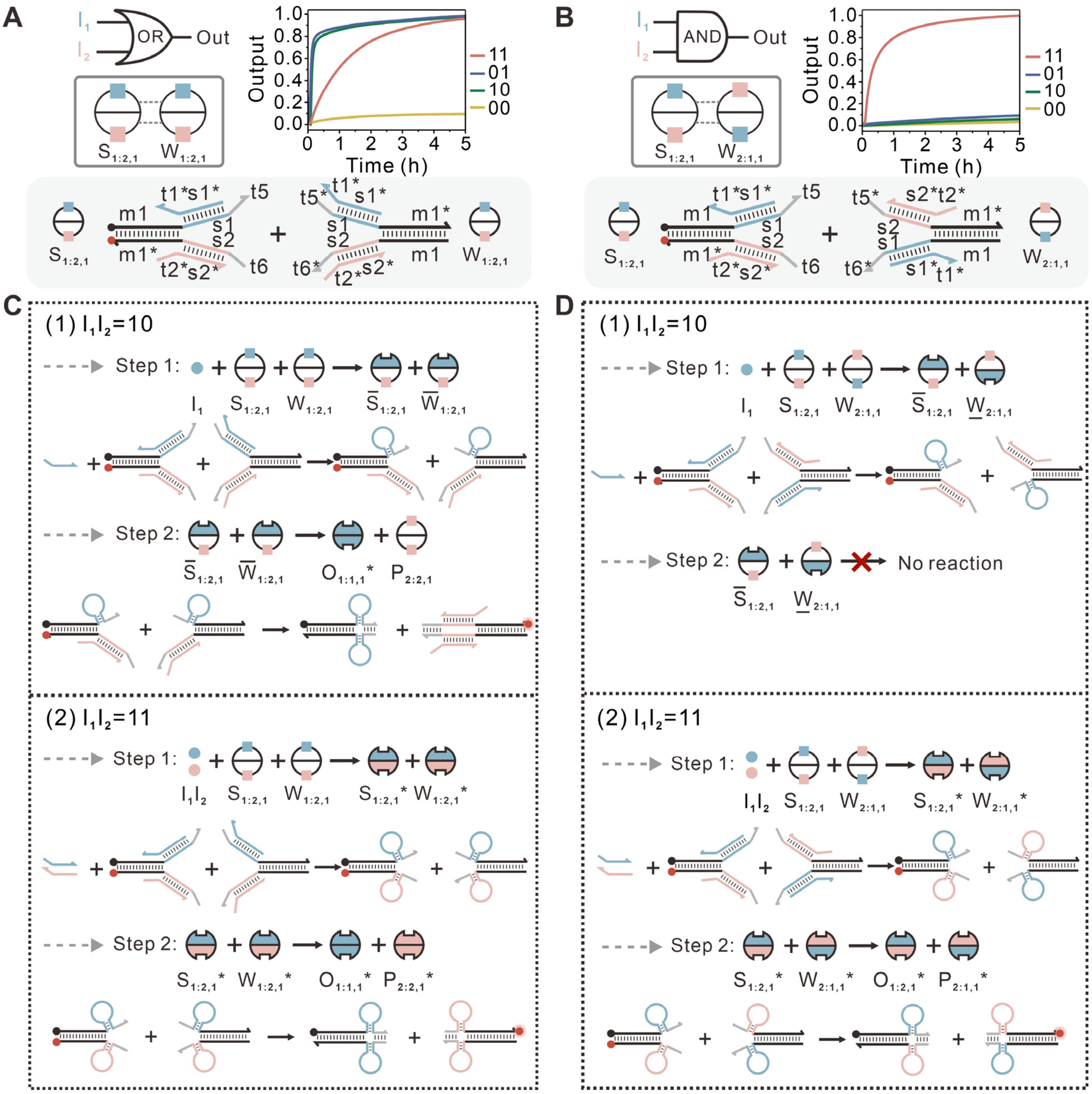

Fig. S10. Two-input ASE-based circuits that compute AND/OR logic. (**A-B**) Schematic representation and its DNA implementation of two ASE-based circuits. Corresponding fluorescence kinetics data are shown on the right. Two distinct molecular switches are carefully distributed at different positions among the two allosteric gates. (**C-D**) Detailed reaction pathways of the ASE-based circuits for different input combinations. Red and black dots denote fluorophore and quencher, respectively. Symbols with subscripts indicate distinct DNA species. The underscore or overline in the symbols indicates the specific position of the activated molecular switch (top or bottom strand) when the gate is in a partially activated state, while symbols marked

with an asterisk signify that all molecular switches within the DNA species are fully activated. The black arrows indicate the flows of the reactions.

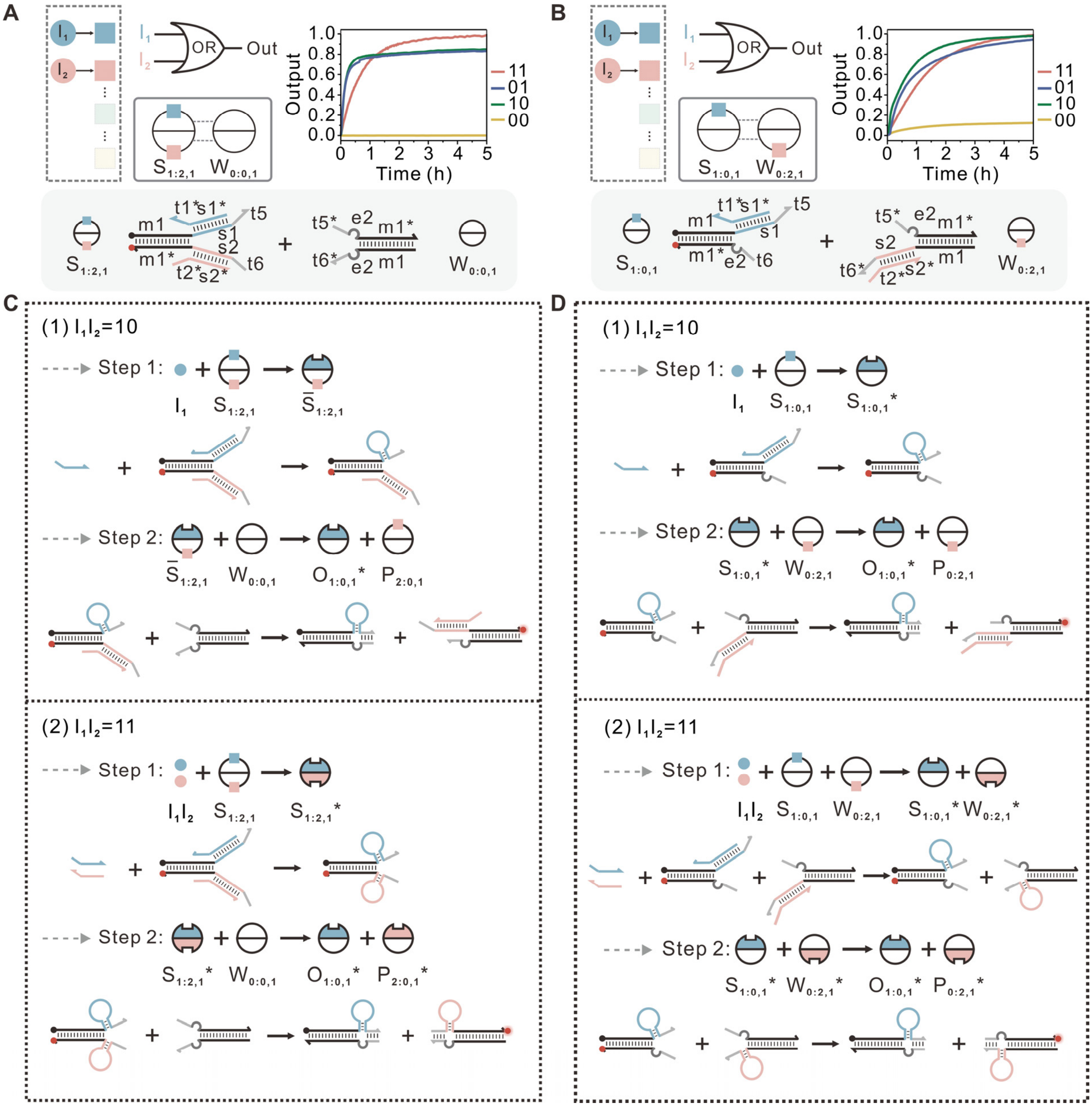

Fig. S11. Different ASE-based circuit configurations for OR logic computation. (**A-B**) Schematic representation and its DNA implementation of two ASE-based circuits. Corresponding fluorescence kinetics data are shown on the right. Two molecular switches with different sequences are carefully distributed at different positions among the two allosteric gates. (**C-D**) Detailed reaction pathways of the ASE-based circuits for different input combinations. Red and black dots denote fluorophore and quencher, respectively. Symbols with subscripts indicate distinct DNA species. The underscore or overline in the symbols indicates the specific position of the activated molecular switch (top or bottom strand) when the gate is in a partially activated state,

while symbols marked with an asterisk signify that all molecular switches within the DNA species are fully activated. The dark gray line indicates a 2-nt spacer inserted between toehold and recognition domains. The black arrows indicate the flows of the reactions.

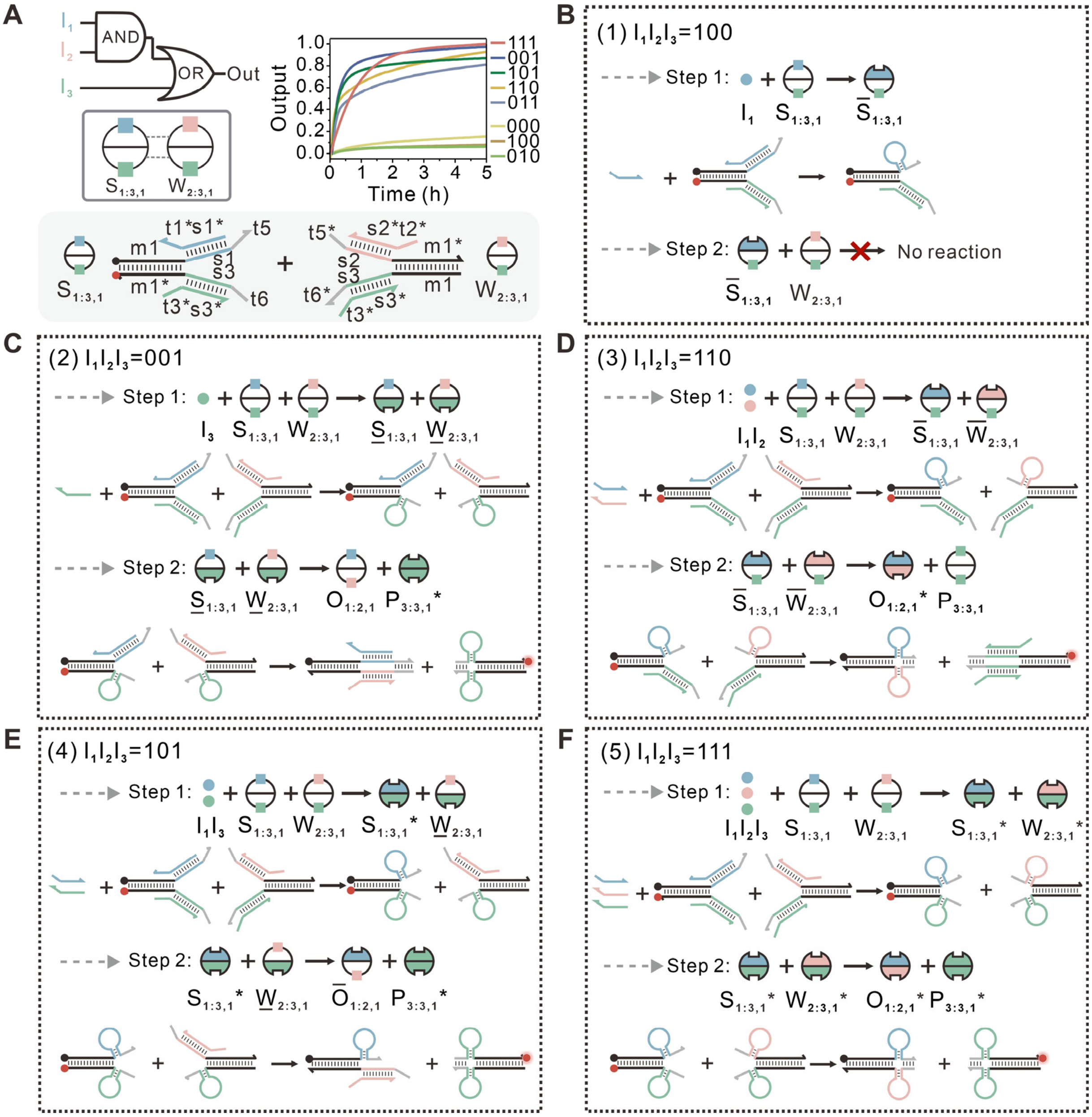

Fig. S12. Three-input ASE-based circuit that computes AND-OR logic computation. (**A**) Schematic representation and its DNA implementation of the three-input ASE-based circuit. Corresponding fluorescence kinetics data are shown on the right. Four molecular switches, two of which maintain the same sequence, are carefully distributed at different positions among allosteric gates. (**B-F**) Detailed reaction pathways of the three-input ASE-based circuit for different input combinations. Red and black dots denote fluorophore and quencher, respectively. Symbols with subscripts indicate distinct DNA species. The underscore or overline in the symbols indicates the specific position of the activated molecular switch (top or bottom strand) when the gate is in a

partially activated state, while symbols marked with an asterisk signify that all molecular switches within the DNA species are fully activated. The black arrows indicate the flows of the reactions.

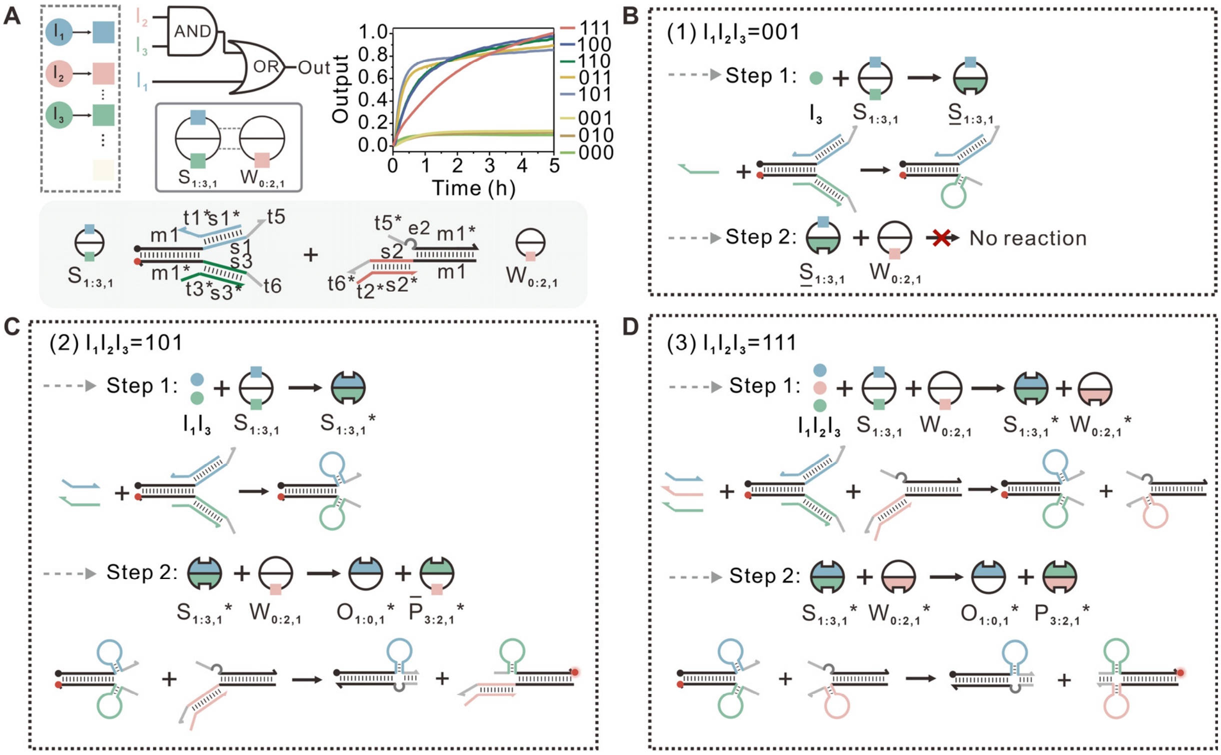

Fig. S13. Three-input ASE-based circuit that computes AND-OR logic computation. (**A**) Schematic representation and its DNA implementation of the three-input ASE-based circuits. Corresponding fluorescence kinetics data are shown on the right. Three molecular switches with different sequences are inserted into two allosteric gates. (**B**-**D**) Detailed reaction pathways of the three-input ASE-based circuit for different input combinations. Red and black dots denote fluorophore and quencher, respectively. Symbols with subscripts indicate distinct DNA species. The underscore or overline in the symbols indicates the specific position of the activated molecular switch (top or bottom strand) when the gate is in a partially activated state, while symbols marked with an asterisk signify that all molecular switches within the DNA species are fully activated. The dark gray line indicates a 2-nt spacer inserted between toehold and recognition domains. The black arrows indicate the flows of the reactions.

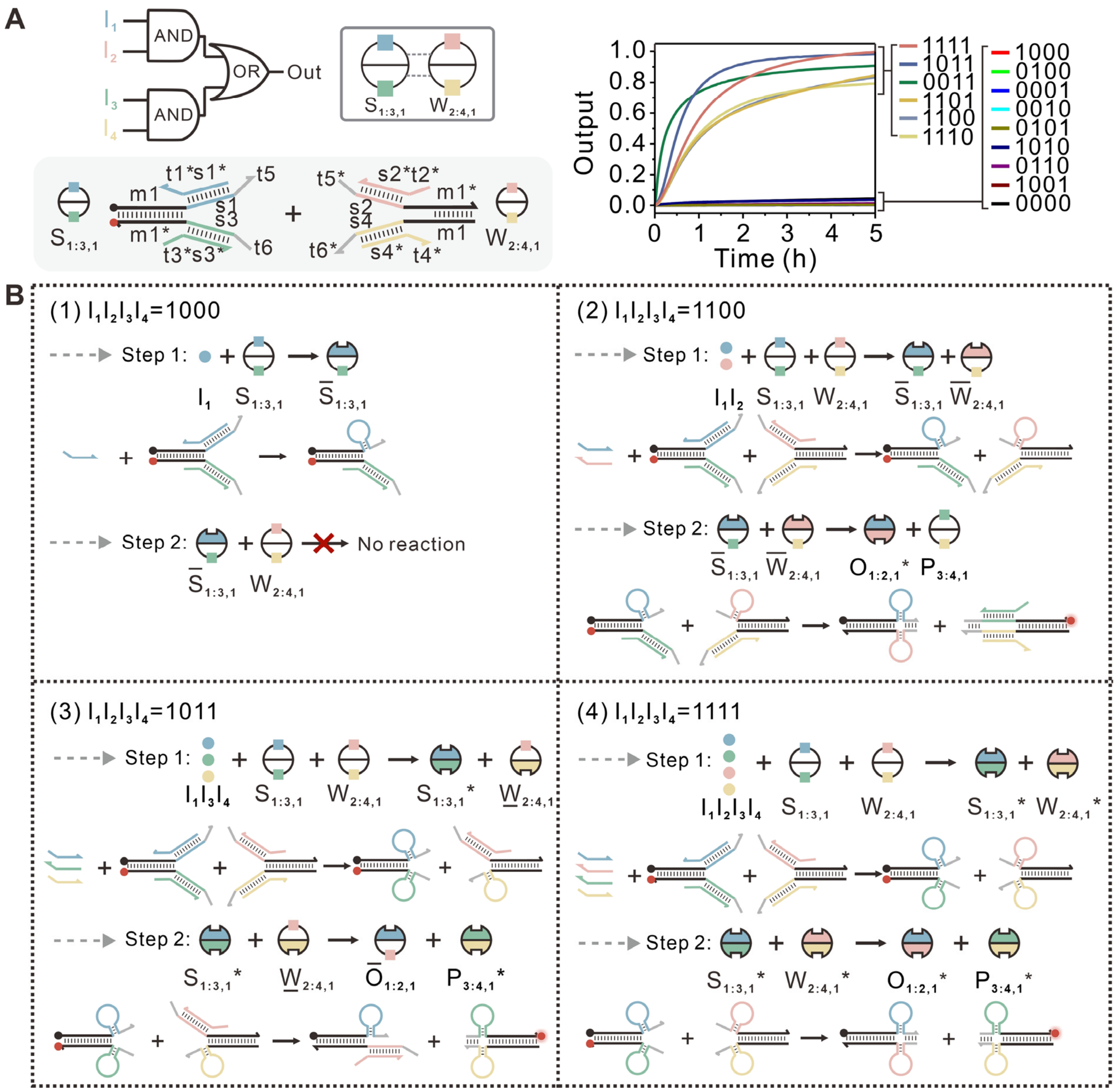

Fig. S14. Four-input ASE-based circuit. (**A**) Schematic representation and its DNA implementation of the four-input ASE-based circuits. Corresponding fluorescence kinetics data are shown on the right. Four molecular switches with different sequences are inserted into two allosteric gates. (**B**) Detailed reaction pathways of the four-input ASE-based circuit for different input combinations. Red and black dots denote fluorophore and quencher, respectively. Symbols with subscripts indicate distinct DNA species. The underscore or overline in the symbols indicates the specific position of the activated molecular switch (top or bottom strand) when the gate is in a

partially activated state, while symbols marked with an asterisk signify that all molecular switches within the DNA species are fully activated. The black arrows indicate the flows of the reactions.

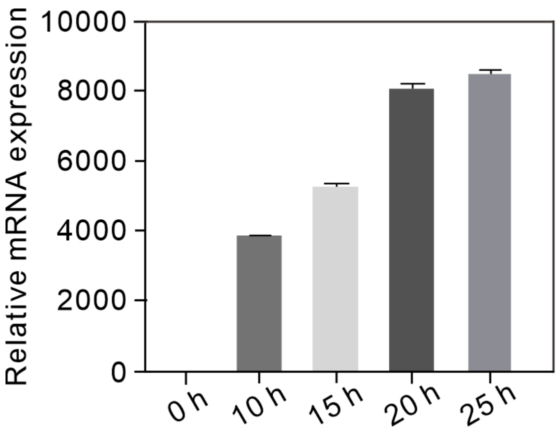

Fig. S15. Relative EGFP mRNA expression at each time point measured by RT-qPCR. HEK293T cells were transfected with the pEGFP-N1 plasmid and incubated for different times. Total RNA was extracted from the cells by Trizol. Error bars indicate standard deviations (mean ± s.d., n = 3). To quantitatively evaluate the performance of the ASE-based circuit in detecting mRNA in mammalian cells, the mRNA copy numbers of target EGFP gene was measured using standard method (*60, 61*) with 18S rRNA as a reference. The experimental data shows that our ASE-based circuit can specifically detect intracellular mRNAs from 9.6×10^3 copies to 2.0×10^4 copies/10^6 18S rRNA copies.

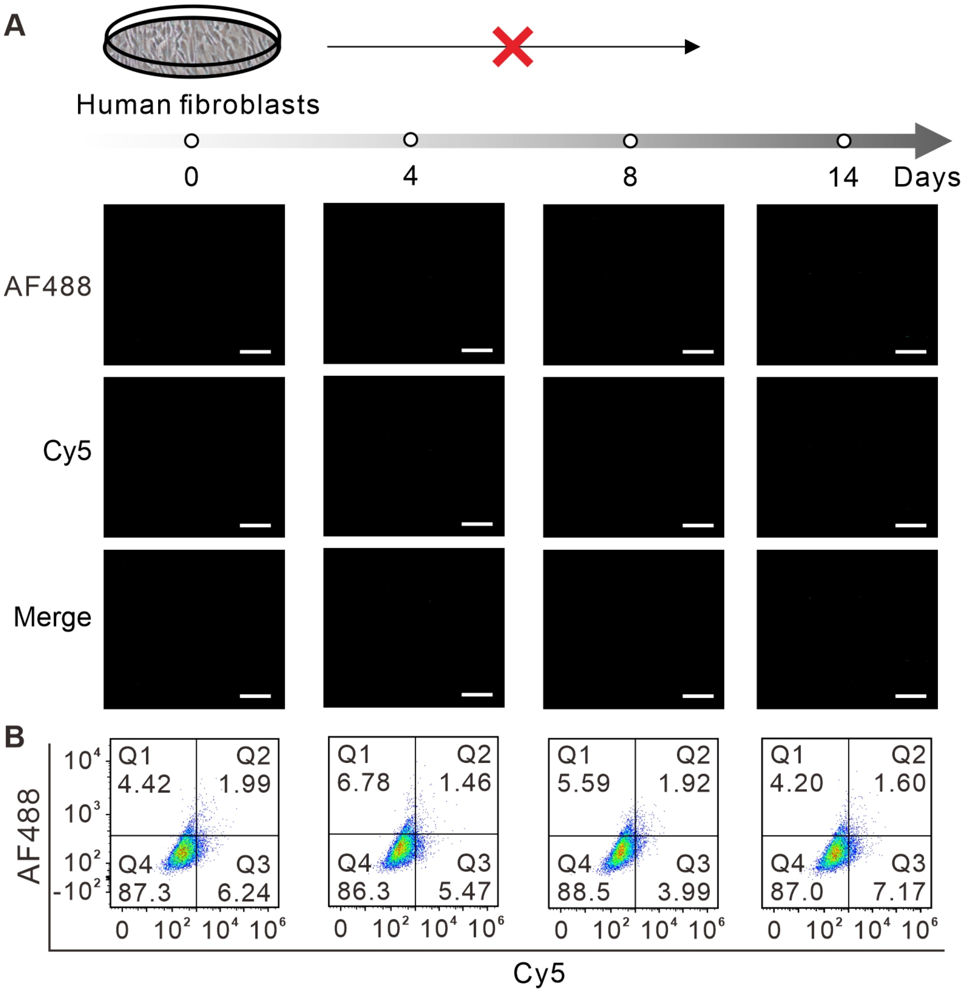

Fig. S16. Control experiments. (**A**) Top: schematic diagram depicting the experimental procedure for the culture of HFFs that did not undergo reprogramming. HFFs were cultured and co-transfected with the classifier circuit as a negative control. Bottom: representative confocal microscopy images showing the behavior of the circuit. All circuit components were co-transfected into the cells at distinct time points. Two fluorophore/quencher pairs were utilized for the characterization of the circuit in cells. Scale bar: 50 µm. (**B**) Flow cytometry results.

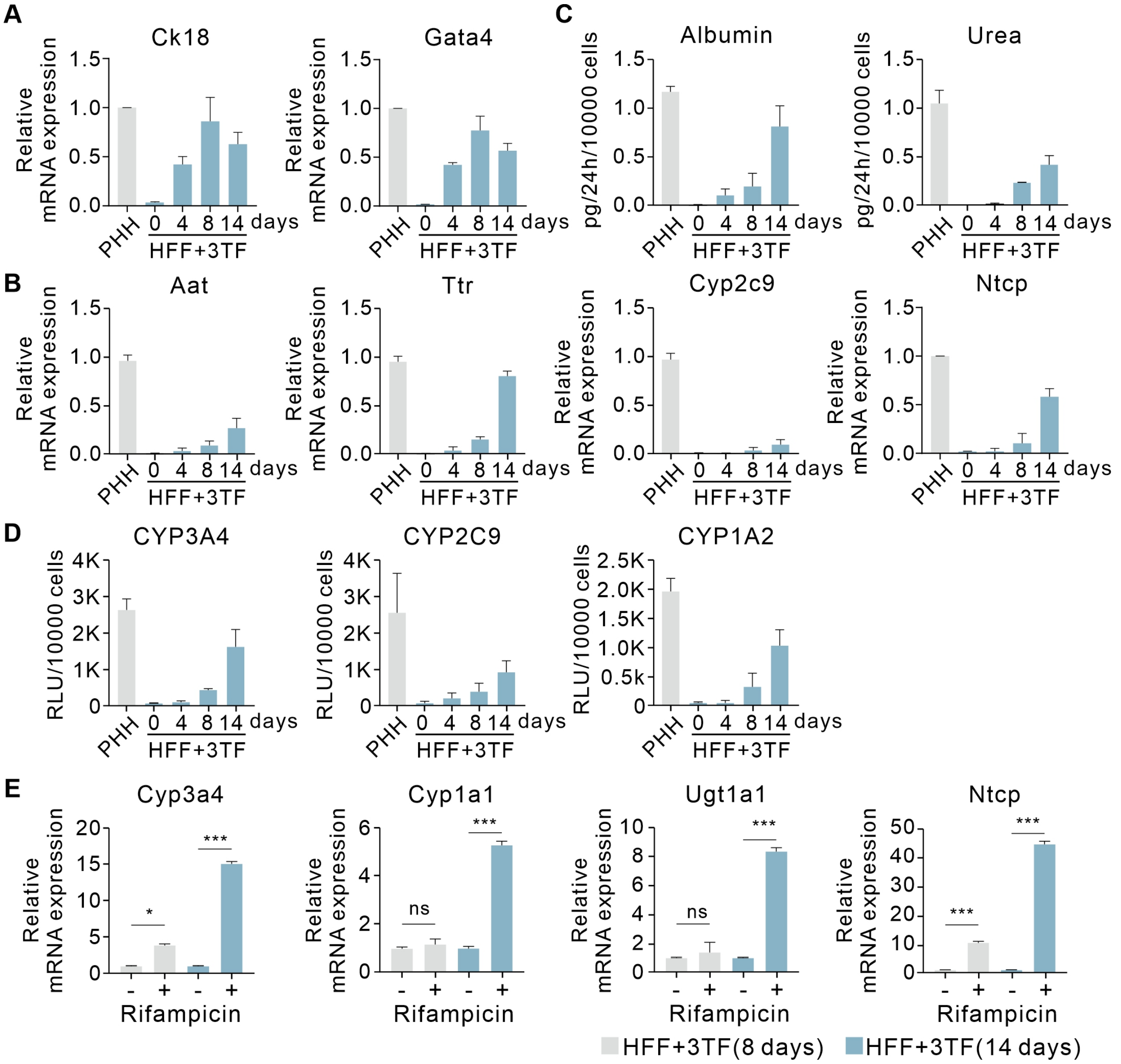

Fig. S17. Hepatic gene expression and functional characterization. (**A-B**) The analysis of relative expression levels for endoderm, epithelial marker genes (**A**) and hepatic genes (**B**) as measured by RT-qPCR. Data were normalized to PHH cultured for 2 days. (**C**) Albumin secretion and Urea synthesis of reprogrammed cells during hepatic conversion. (**D**) CYP activity assay. PHHs cultured for 2 days were used as a positive control. (**E**) CYP metabolism assay. Elevated levels of CYP3A4, CYP1A1, UGT1A1, and NTCP mRNA were measured by RT-qPCR. Error bars indicate standard deviations (mean ± s.d., n = 3).

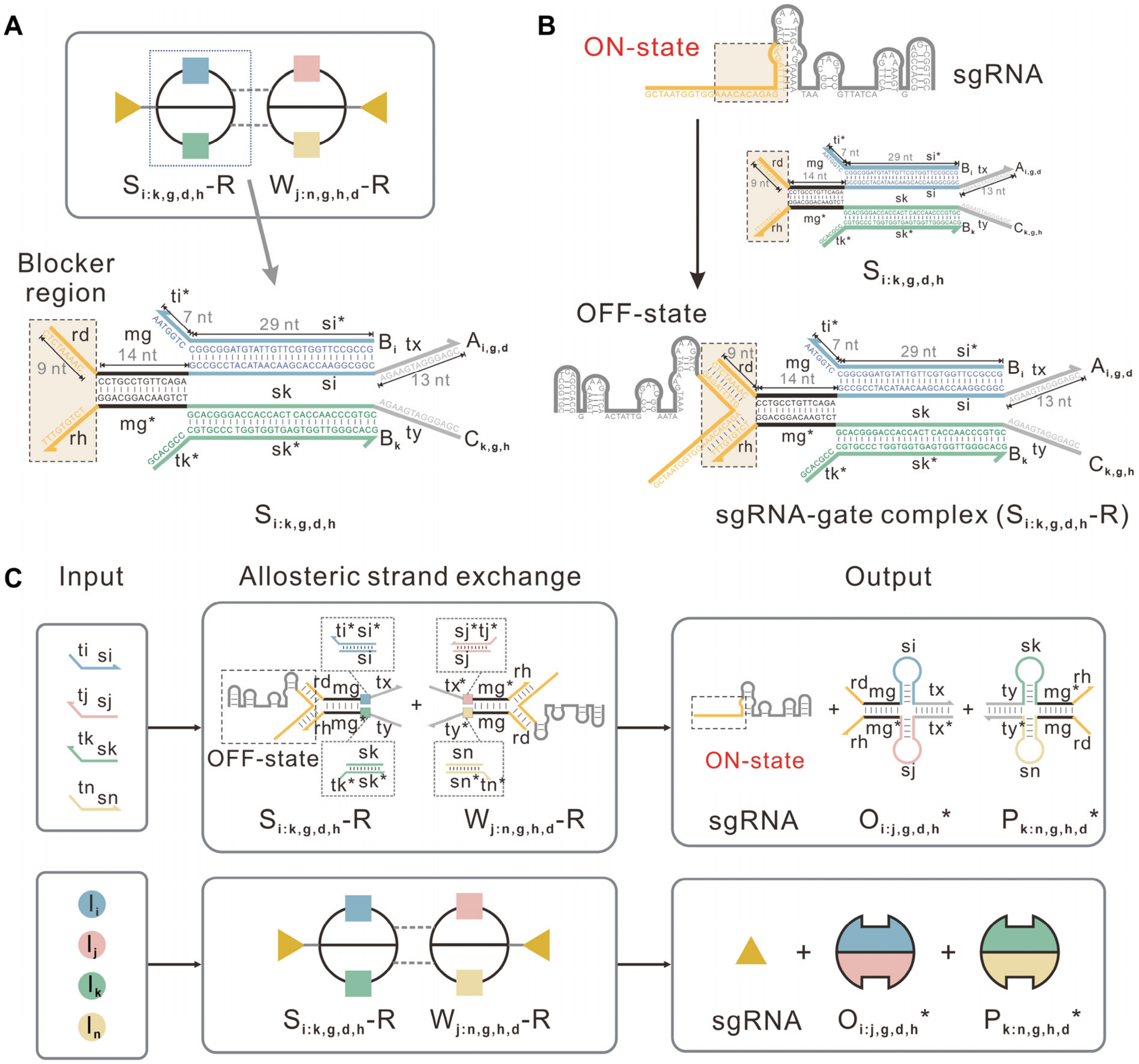

Fig. S18. Reprogramming the ASE-based circuit for integration with CRISPR-Cas9. (**A**) Each allosteric gate is redesigned to incorporate a 9-nt blocker region (highlighted by yellow lines). (**B**) Schematic of sgRNA-gate complex assembling process. sgRNA is hybridized to the blocker regions of the allosteric gate to form sgRNA-gate complex, inducing a conformational change that switches the sgRNA to an OFF state and inhibits its target recognizing activity. (**C**) Schematic of the sgRNA activation by ASE-based circuit. The originally bound sgRNA can be released upon the arrival of corresponding input strands, and then restore its ON state activity. Symbols with subscripts indicate distinct oligonucleotide species, with asterisks in the names indicating that all molecular switches within gates are activated. The black arrows indicate the flows of the reactions.

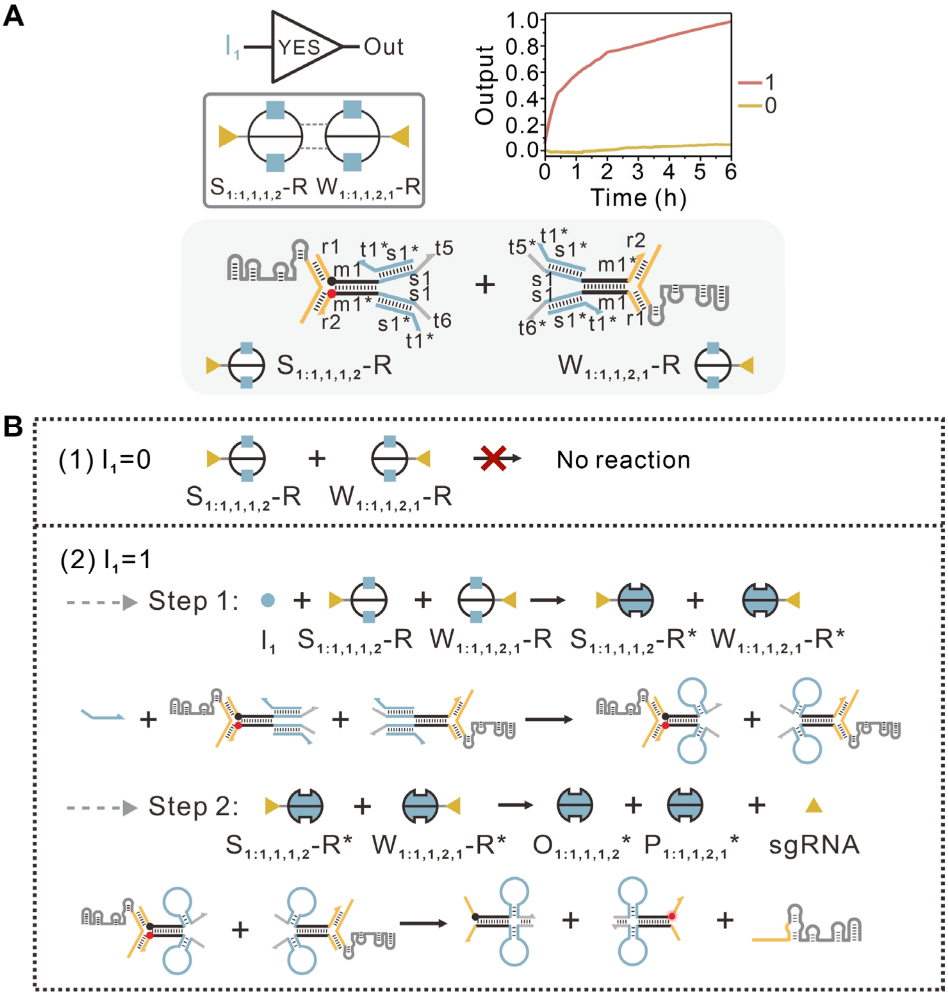

Fig. S19. Implementation of the ASE-based circuit with YES logic operation for sgRNA activation. (**A**) Design schematic and its DNA implementation of ASE-based circuit. The fluorescence kinetics data is shown on the right. Four molecular switches with the same sequence are inserted into the allosteric gates to build the circuit that compute YES logic function. (**B**) Detailed reaction pathways of the circuit in the absence or presence of input. Red and black dots denote fluorophore and quencher, respectively. Symbols with subscripts indicate distinct oligonucleotide species, with asterisks in the names indicating that all molecular switches within gates are activated. The black arrows indicate the flows of the reactions.

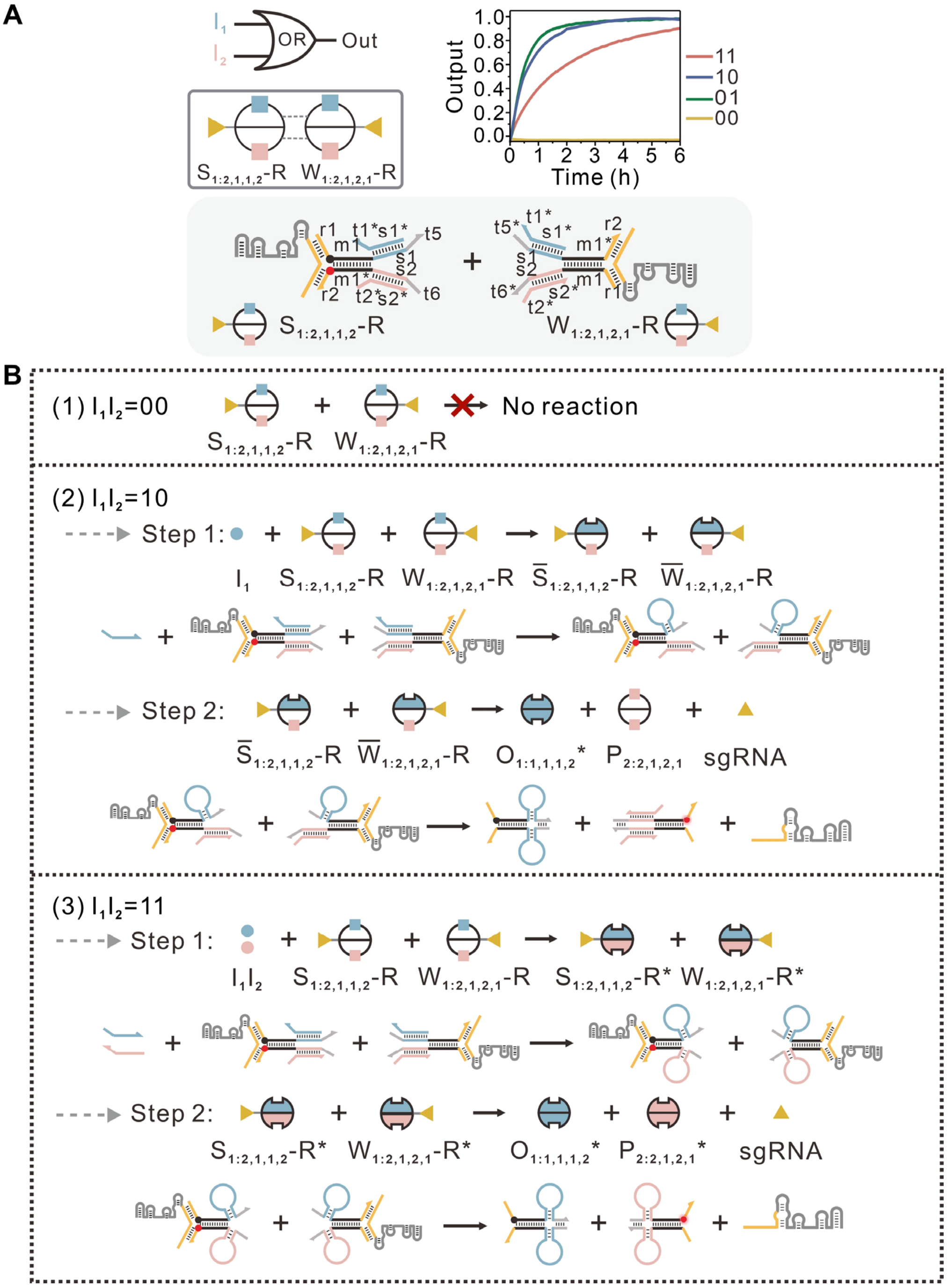

Fig. S20. Implementation of the two-input ASE-based circuit with OR logic operation for sgRNA activation. (**A**) Design schematic and its DNA implementation of ASE-based circuit. The fluorescence kinetics data is shown on the right. Two distinct molecular switches are inserted at specific positions to build the circuit that compute OR logic function. (**B**) Detailed reaction pathways of the circuit for different input combinations. Red and black dots denote fluorophore

and quencher, respectively. Symbols with subscripts indicate distinct DNA species. The underscore or overline in the symbols indicates the specific position of the activated molecular switch (top or bottom strand) when the gate is in a partially activated state, while symbols marked with an asterisk signify that all molecular switches within the DNA species are fully activated. The black arrows indicate the flows of the reactions.

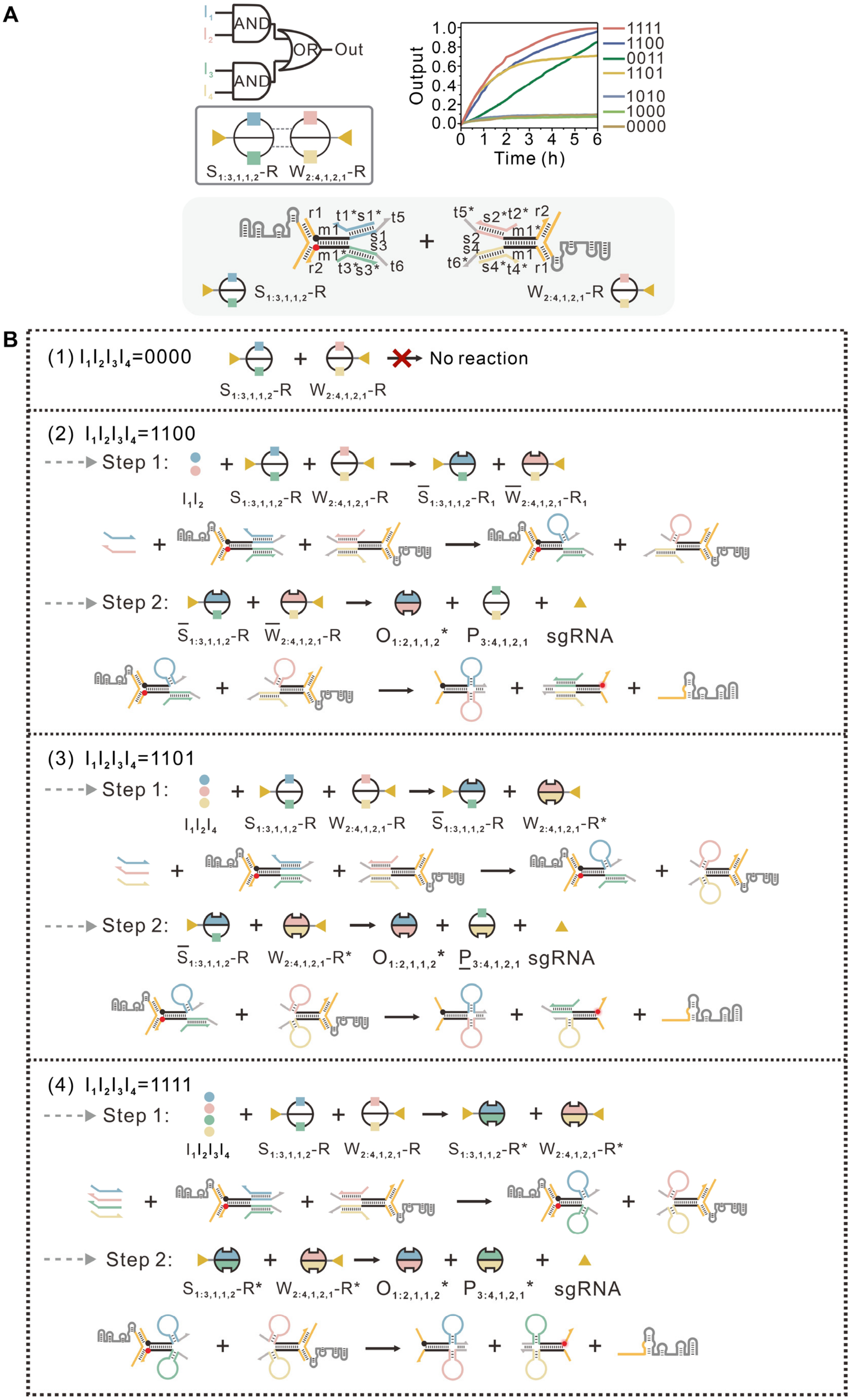

A
AND
AND
OR
Out
S1:3,1,1,2-R
W2:4,1,2,1-R
Output
Time (h)
1111
1100
0011
1101
1010
1000
0000
r1
t1*s1*
t5
m1
s1
s3
m1*
r2
t3*s3*
t6
t5*
s2*t2*
r2
s2
s4
t6*
s4*t4*
r1
S1:3,1,1,2-R
W2:4,1,2,1-R
B
(1) I1I2I3I4=0000
No reaction
S1:3,1,1,2-R
W2:4,1,2,1-R
(2) I1I2I3I4=1100
Step 1:
I1I2
S1:3,1,1,2-R
W2:4,1,2,1-R
S1:3,1,1,2-R1
W2:4,1,2,1-R1
Step 2:
S1:3,1,1,2-R
W2:4,1,2,1-R
O1:2,1,1,2*
P3:4,1,2,1
sgRNA
(3) I1I2I3I4=1101
Step 1:
I1I2I4
S1:3,1,1,2-R
W2:4,1,2,1-R
S1:3,1,1,2-R
W2:4,1,2,1-R*
Step 2:
S1:3,1,1,2-R
W2:4,1,2,1-R*
O1:2,1,1,2*
P3:4,1,2,1
sgRNA
(4) I1I2I3I4=1111
Step 1:
I1I2I3I4
S1:3,1,1,2-R
W2:4,1,2,1-R
S1:3,1,1,2-R*
W2:4,1,2,1-R*
Step 2:
S1:3,1,1,2-R*
W2:4,1,2,1-R*
O1:2,1,1,2*
P3:4,1,2,1*
sgRNA

Fig. S21. Implementation of the four-input ASE-based circuit for sgRNA activation. (**A**) Design schematic and its DNA implementation of ASE-based circuit. The fluorescence kinetics data is shown on the right. Four distinct molecular switches are inserted into the allosteric gates to build the circuit that compute a four-input logic expression. (**B**) Detailed reaction pathways of the circuit for different input combinations. Red and black dots denote fluorophore and quencher, respectively. Symbols with subscripts indicate distinct DNA species. The underscore or overline in the symbols indicates the specific position of the activated molecular switch (top or bottom strand) when the gate is in a partially activated state, while symbols marked with an asterisk signify that all molecular switches within the DNA species are fully activated. The black arrows indicate the flows of the reactions.

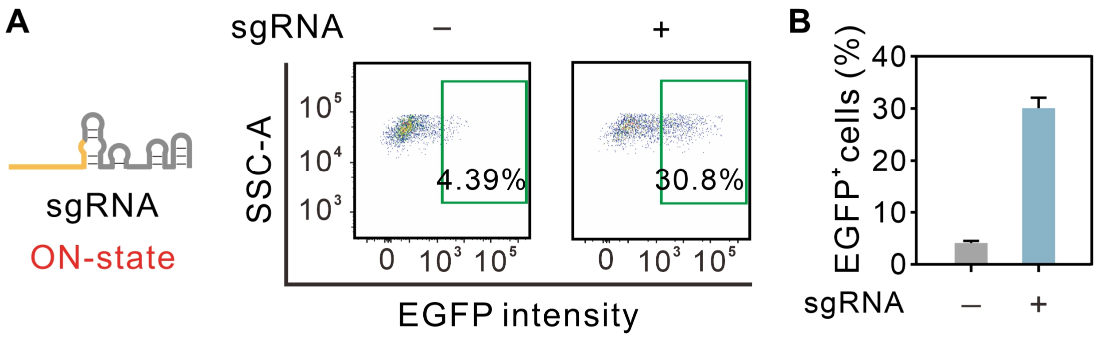

Fig. S22. Experimental data showing the target binding efficiency with ON-state sgRNA itself. (**A-B**) Flow cytometry analysis of HEK293T cells treated with or without ON-state sgRNA. HEK293T cells were first transfected with Cas9 and a deactivated EGFP gene disrupted by the insertion of a stop codon and a target genomic sequence. Twenty-four hours later, the cells were transfected with ON-state sgRNA. The ratio of EGFP-positive cells was measured by flow cytometry. Error bars indicate standard deviations (mean ± s.d., n = 3). The results demonstrated that the ON-state sgRNA itself induced a ~30% increase in fluorescent cells (EGFP positive). As shown in Fig. 5**B**-5**D**, the ASE-based Cas9 activation led to a ~17% to 21% increase in fluorescent cells (EGFP positive), which was slightly lower than the effect observed with the ON-state sgRNA itself. This discrepancy may be attributed to incomplete circuit activation, potentially due to the uneven distribution of inputs and sgRNA-gate complexes among cells. Consequently, in many cells, the input concentration might have been insufficient to fully activate all sgRNA-gate complexes.

Table S1. Strand sequences for mechanism investigation of ASE in Fig. 1 and Fig. S2-5.

Strand Name	Sequence (from 5' to 3')
Dabcyl-$A_{1,1}$	/Dabcyl/ CCTGCCTGTTCAGA GCCGCCTACATAACAAGCACCAAGGC GGC AGAAGTAGGGAGC
$A_{0,1}$ (no spacer)	CCTGCCTGTTCAGA TCTTCATCCCTCG
$A_{0,1}$ ('TT' spacer)	CCTGCCTGTTCAGA TT TCTTCATCCCTCG
$A_{1,1}$	CCTGCCTGTTCAGA GCCGCCTACATAACAAGCACCAAGGCGGC TC TTCATCCCTCG
$C_{0,1}$ (no spacer)	GCTCCCTACTTCT TCTGAACAGGCAGG
$C_{0,1}$ ('TT' spacer)	GCTCCCTACTTCT TT TCTGAACAGGCAGG
$C_{1,1}$	GCTCCCTACTTCT GCCGCCTACATAACAAGCACCAAGGCGGC TCT GAACAGGCAGG
$C_{0,1}$ (no spacer)-FAM	CGAGGGATGAAGA TCTGAACAGGCAGG /FAM/
$C_{0,1}$ ('TT' spacer)-FAM	CGAGGGATGAAGA TT TCTGAACAGGCAGG /FAM/
$C_{1,1}$-FAM	CGAGGGATGAAGA GCCGCCTACATAACAAGCACCAAGGCGGC TCT GAACAGGCAGG /FAM/
B_1	GCCGCCTTGGTGCTTGTTATGTAGGCGGC CTGGTAA
I_1	TTACCAG GCCGCCTACATAACAAGCACCAAGGCGGC

Table S2. Strand sequences for in-cell characterization of ASE in Fig. S7-9.

Strand Name	Sequence (from 5' to 3')
$A_{1,1}$ (DNA)	CCTGCCTGTTCAGA GCCGCCTACATAACAAGCACCAAGGCGGC AGAAGTAGGGAGC
BHQ1-$A_{1,1}$ (DNA)	/BHQ1/ CCTGCCTGTTCAGA GCCGCCTACATAACAAGCACCAAGG CGGC AGAAGTAGGGAGC
$C_{1,1}$-AF488 (DNA)	CGAGGGATGAAGA GCCGCCTACATAACAAGCACCAAGGCGGC T CTGAACAGGCAGG /AF488/
I_1-Cy5 (DNA)	TTACCAG GCCGCCTACATAACAAGCACCAAGGCGGC /Cy5/
I_x (DNA)	GCCGCCAAGTGAGGTAGAGTAGTGGCGGCTCAACGG
BHQ3-$A_{1,1}$ (2′OMe RNA)	/BHQ3/ mCmCmUmGmCmCmUmGmUmUmCmAmGmA mGmCmCm GmCmCmUmAmCmAmUmAmAmCmAmAmGmCmAmCmCmAmAm GmGmCmGmGmC mAmGmAmAmGmUmAmGmGmGmAmGmC
$A_{1,1}$ (2′OMe RNA)	mCmCmUmGmCmCmUmGmUmUmCmAmGmA mGmCmCmGmCmC mUmAmCmAmUmAmAmCmAmAmGmCmAmCmCmAmAmGmGmC mGmGmC mUmCmUmUmCmAmUmCmCmCmUmCmG
$C_{1,1}$ (2′OMe RNA)	mGmCmUmCmCmCmUmAmCmUmUmCmU mGmCmCmGmCmCmU mAmCmAmUmAmAmCmAmAmGmCmAmCmCmAmAmGmGmCmG mGmC mUmCmUmGmAmAmCmAmGmGmCmAmGmG
$C_{1,1}$ (2′OMe RNA)-Cy5	mCmGmAmGmGmGmAmUmGmAmAmGmA mGmCmCmGmCmCmU mAmCmAmUmAmAmCmAmAmGmCmAmCmCmAmAmGmGmCmG mGmCm mUmCmUmGmAmAmCmAmGmGmCmAmGmG /Cy5/
B_1 (2′OMe RNA)	mGmCmCmGmCmCmUmUmGmGmUmGmCmUmUmGmUmUmAmU mGmUmAmGmGmCmGmGmC mCmUmGmGmUmAmA
I_1 (2′OMe RNA)	mUmUmAmCmCmAmG mGmCmCmGmCmCmUmAmCmAmUmAmA mCmAmAmGmCmAmCmCmAmAmGmGmCmGmGmC

Table S3. Strand sequences for ASE-based circuits in Fig. 2 and Fig. S10-14.

Strand Name	Sequence (from 5' to 3')
Dabcyl-$A_{5,2}$	/Dabcyl/ AGACTTGTCCGTCC ACGTCAGATTACTGCAACTATCCTGACGT A GAAGTAGGGAGC
$A_{2,1}$	CCTGCCTGTTCAGA CGGCGGTTCACTCCATCTCATCACCGCCG TCTTCAT CCCTCG
$A_{3,1}$	CCTGCCTGTTCAGA CGTGCCCAACCACTCACCACCAGGGCACG TCTTCA TCCCTCG
$A_{4,1}$	CCTGCCTGTTCAGA GCACGCCTTATTATTATCTCTTGGCGTGC TCTTCATC CCTCG
$A_{8,2}$	AGACTTGTCCGTCC GCTCTGCCAAGTATGAGGAGCTGCAGAGC TCTTCA TCCCTCG
$C_{2,1}$	GCTCCCTACTTCT CGGCGGTTCACTCCATCTCATCACCGCCG TCTGAACA GGCAGG
$C_{6,2}$	GCTCCCTACTTCT GGAGCAGATCAAGACCCTCAACCTGCTCC GGACGGA CAAGTCT
$C_{2,1}$-FAM	CGAGGGATGAAGA CGGCGGTTCACTCCATCTCATCACCGCCG TCTGAAC AGGCAGG /FAM/
$C_{3,1}$-FAM	CGAGGGATGAAGA CGTGCCCAACCACTCACCACCAGGGCACG TCTGAA CAGGCAGG /FAM/
$C_{7,2}$-FAM	CGAGGGATGAAGA GACCCGGCTTTCTTCGCCGCCCCCGGGTC GGACGG ACAAGTCT /FAM/
B_2	GGCAAGT CGGCGGTGATGAGATGGAGTGAACCGCCG
B_3	TCTCACT CGTGCCCTGGTGGTGAGTGGTTGGGCACG
B_4	GCACGCCAAGAGATAATAATAAGGCGTGC AAGATCC
B_5	ACGTCAGGATAGTTGCAGTAATCTGACGT ATACCGC
B_6	ACTTGTT GGAGCAGGTTGAGGGTCTTGATCTGCTCC
B_7	GGCATCG GACCCGGGGGCGGCGAAGAAAGCCGGGTC
B_8	GCTCTGCAGCTCCTCATACTTGGCAGAGC ATCTGGT
I_2	CGGCGGTTCACTCCATCTCATCACCGCCG ACTTGCC
I_3	CGTGCCCAACCACTCACCACCAGGGCACG AGUGAGA

I_4	GGATCTT GCACGCCTTATTATTATCTCTTGGCGTGC
I_5	GCGGTAT ACGTCAGATTACTGCAACTATCCTGACGT
I_6	GGAGCAGATCAAGACCCTCAACCTGCTCC AACAAGT
I_7	GACCCGGCTTTCTTCGCCGCCCCCGGGTC CGATGCC
I_8	ACCAGAT GCTCTGCCAAGTATGAGGAGCTGCAGAGC

Table S4. Strand sequences for ASE-based intracellular AND logic in Fig. 3.

**Strand Name**	**Sequence (from 5’ to 3’)**
BHQ3-$A_{1,1}$ (2′OMe RNA)	/BHQ3/ mCmCmUmGmCmCmUmGmUmUmCmAmGmA mUmCmGm<br>UmCmCmUmGmCmAmCmCmAmUmCmUmUmCmUmUmCmAmAm<br>GmGmAmCmGmA mAmGmAmAmGmUmAmGmGmGmAmGmC
$A_{1,1}$ (2′OMe RNA)	mCmCmUmGmCmCmUmGmUmUmCmAmGmA mCmAmGmGmAmG<br>mCmGmCmAmCmCmAmUmCmUmUmCmUmUmCmAmGmCmUmC<br>mCmUmG mUmCmUmUmCmAmUmCmCmCmUmCmG
$C_{2,1}$ (2′OMe RNA)	mGmCmUmCmCmCmUmAmCmUmUmCmU mAmCmUmAmCmCmU<br>mGmAmGmCmAmCmCmCmAmGmUmCmCmGmCmAmGmGmUmA<br>mGmU mUmCmUmGmAmAmCmAmGmGmCmAmGmG
$C_{2,1}$-Cy5 (2′OMe RNA)	mCmGmAmGmGmGmAmUmGmAmAmGmA mGmCmUmCmAmGmG<br>mGmAmGmCmAmCmCmCmAmGmUmCmCmGmCmCmCmUmGmA<br>mGmC mUmCmUmGmAmAmCmAmGmGmCmAmGmG /Cy5/
B_1 (2′OMe RNA)	mUmCmGmUmCmCmUmUmGmAmAmGmAmAmGmAmUmGmGmU<br>mGmC mGmCmUmCmCmUmG
B_2 (2′OMe RNA)	mGmCmUmCmAmGmG mGmCmGmGmAmCmUmGmGmGmUmGmC<br>mUmCmAmGmGmUmAmGmU
MB (2′OMe RNA)	mUmCmGmUmCmCmU mUmGmAmAmGmAmAmGmAmUmGmGmU<br>mGmC mAmGmGmAmCmGmA

Table S5. Strand sequences for ASE-based intracellular multi-input molecular classifier in Fig. 4 and Fig. S16.

Strand Name	Sequence (from 5’ to 3’)
BHQ1-$A_{1,1}$ (2′OMe RNA)	/BHQ1/ mCmCmUmGmCmCmUmGmUmUmCmAmGmA mAmCmGm UmCmAmGmAmUmUmAmCmUmGmCmAmAmCmUmAmUmCmCm UmGmAmCmGmU mAmGmAmAmGmUmAmGmGmGmAmGmC
$A_{4,1}$ (2′OMe RNA)	mCmCmUmGmCmCmUmGmUmUmCmAmGmA mGmAmCmCmCmG mGmCmUmUmUmCmUmUmCmGmCmCmGmCmCmCmCmGmG mGmUmC mUmCmUmUmCmAmUmCmCmCmUmCmG
$C_{2,1}$ (2′OMe RNA)	mGmCmUmCmCmCmUmAmCmUmUmCmU mGmGmAmGmCmAmG mAmUmCmAmAmGmAmCmCmCmUmCmAmAmCmCmUmGmCmU mCmC mUmCmUmGmAmAmCmAmGmGmCmAmGmG
$C_{3,1}$-AF488 (2′OMe RNA)	mCmGmAmGmGmGmAmUmGmAmAmGmA mGmCmUmCmUmGmC mCmAmAmGmUmAmUmGmAmGmGmAmGmCmUmGmCmAmGmA mGmC mUmCmUmGmAmAmCmAmGmGmCmAmGmG /AF488/
BHQ3-$A_{5,2}$ (2′OMe RNA)	/BHQ3/ mAmGmAmCmUmUmGmUmCmCmGmUmCmC mGmGmAm GmGmUmUmAmAmAmGmAmUmGmAmCmAmAmCmCmCmAmAm AmCmCmUmCmC mCmGmAmGmGmGmAmUmGmAmAmGmA
$A_{8,2}$ (2′OMe RNA)	mAmGmAmCmUmUmGmUmCmCmGmUmCmC mCmCmAmUmGmC mAmGmAmUmAmUmAmUmGmCmAmCmAmCmUmUmUmGmCmA mUmGmG mGmCmUmCmCmCmUmAmCmUmUmCmU
$C_{6,2}$ (2′OMe RNA)	mUmCmUmUmCmAmUmCmCmCmUmCmG mCmUmAmUmAmGmC mUmGmAmGmGmAmUmGmAmAmGmAmAmUmGmGmCmUmAmU mAmG mGmGmAmCmGmGmAmCmAmAmGmUmCmU
$C_{7,2}$-Cy5 (2′OMe RNA)	mAmGmAmAmGmUmAmGmGmGmAmGmC mGmAmCmGmUmCmU mGmCmCmUmGmUmCmAmCmCmUmUmGmAmAmAmGmAmCmG mUmC mGmGmAmCmGmGmAmCmAmAmGmUmCmU /Cy5/
B_1 (2′OMe RNA)	mAmCmGmUmCmAmGmGmAmUmAmGmUmUmGmCmAmGmUmA mAmU mAmUmAmCmCmGmC
B_2 (2′OMe RNA)	mAmCmUmUmGmUmU mGmUmUmGmAmGmGmGmUmCmUmUmG mAmUmCmUmGmCmUmCmC

B_3 (2′OMe RNA)	mGmCmUmCmUmGmCmAmGmCmUmCmCmUmCmAmUmAmCmU mUmG mAmUmCmUmGmGmU
B_4 (2′OMe RNA)	mGmGmCmAmUmCmG mGmGmGmCmGmGmCmGmAmAmGmAmA mAmGmCmCmGmGmGmUmC
B_5 (2′OMe RNA)	mGmGmAmGmGmUmUmUmGmGmGmUmUmGmUmCmAmUmCmU mUmUmGmUmGmUmUmGmC
B_6 (2′OMe RNA)	mUmCmUmCmUmUmCmCmAmUmUmCmUmUmCmAmUmCmCmU mCmAmGmCmUmAmUmAmG
B_7 (2′OMe RNA)	mGmAmCmGmUmCmUmUmUmCmAmAmGmGmUmGmAmCmAmG mGmCmUmUmGmCmCmUmG
B_8 (2′OMe RNA)	mUmCmUmCmAmGmAmAmAmGmUmGmUmGmCmAmUmAmUmA mUmCmUmGmCmAmUmGmG

Table S6. Strand sequences for ASE-based intracellular circuit interfaced with the CRISPR-Cas9 in Fig. 5 and Fig. S19-22.

Strand Name	Sequence (from 5' to 3')
BHQ3-$A_{1,1,1}$ (2′OMe RNA)	mCmUmCmUmAmAmAmAmC /BHQ3/ mCmCmUmGmCmCmUmGm UmUmCmAmGmA mGmCmCmGmCmCmUmAmCmAmUmAmAmC mAmAmGmCmAmCmCmAmAmGmGmCmGmGmC mAmGmAmAm GmUmAmGmGmGmAmGmC
$A_{1,1,1}$ (2′OMe RNA)	mCmUmCmUmAmAmAmAmC mCmCmUmGmCmCmUmGmUmUmC mAmGmA mGmCmCmGmCmCmUmAmCmAmUmAmAmCmAmAm GmCmAmCmCmAmAmGmGmCmGmGmC mUmCmUmUmCmAmU mCmCmCmUmCmG
$A_{2,1,1}$ (2′OMe RNA)	mCmUmCmUmAmAmAmAmC mCmCmUmGmCmCmUmGmUmUmC mAmGmA mCmGmGmCmGmGmUmUmCmAmCmUmCmCmAmUm CmUmCmAmUmCmAmCmCmGmCmCmG mUmCmUmUmCmAmUm CmCmCmUmCmG
$A_{4,1,1}$ (2′OMe RNA)	mCmUmCmUmAmAmAmAmC mCmCmUmGmCmCmUmGmUmUmC mAmGmA mGmCmAmCmGmCmCmUmUmAmUmUmAmUmUmAm UmCmUmCmUmUmGmGmCmGmUmGmC mUmCmUmUmCmAmU mCmCmCmUmCmG
$C_{1,1,2}$ (2′OMe RNA)	mGmCmUmCmCmCmUmAmCmUmUmCmU mGmCmCmGmCmCmU mAmCmAmUmAmAmCmAmAmGmCmAmCmCmAmAmGmGmCmG mGmC mUmCmUmGmAmAmCmAmGmGmCmAmGmG mUmCmUm GmUmGmUmUmU
$C_{2,1,2}$ (2′OMe RNA)	mGmCmUmCmCmCmUmAmCmUmUmCmU mCmGmGmCmGmGmU mUmCmAmCmUmCmCmAmUmCmUmCmAmUmCmAmCmCmGmC mCmG mUmCmUmGmAmAmCmAmGmGmCmAmGmG mUmCmUm GmUmGmUmUmU
$C_{1,1,2}$-Cy5 (2′OMe RNA)	mCmGmAmGmGmGmAmUmGmAmAmGmA mGmCmCmGmCmCm UmAmCmAmUmAmAmCmAmAmGmCmAmCmCmAmAmGmGmCm GmGmC mUmCmUmGmAmAmCmAmGmGmCmAmGmG /Cy5/ mUm CmUmGmUmGmUmUmU

$C_{2,1,2}$-Cy5 (2′OMe RNA)	mCmGmAmGmGmGmAmUmGmAmAmGmA mCmGmGmCmGmGm UmUmCmAmCmUmCmCmAmUmCmUmCmAmUmCmAmCmCmGm CmCmG mUmCmUmGmAmAmCmAmGmGmCmAmGmG /Cy5/ mUm CmUmGmUmGmUmUmU
$C_{3,1,2}$-Cy5 (2′OMe RNA)	mCmGmAmGmGmGmAmUmGmAmAmGmA mCmGmUmGmCmCm CmAmAmCmCmAmCmUmCmAmCmCmAmCmCmAmGmGmGmCm AmCmG mUmCmUmGmAmAmCmAmGmGmCmAmGmG /Cy5/ mUm CmUmGmUmGmUmUmU
B_2 (2′OMe RNA)	mGmGmCmAmAmGmU mCmGmGmCmGmGmUmGmAmUmGmAm GmAmUmGmGmAmGmUmGmAmAmCmCmGmCmCmG
B_3 (2′OMe RNA)	mUmCmUmCmAmCmU mCmGmUmGmCmCmCmUmGmGmUmGm GmUmGmAmGmUmGmGmUmUmGmGmGmCmAmCmG
B_4 (2′OMe RNA)	mGmCmAmCmGmCmCmAmAmGmAmGmAmUmAmAmUmAmAmU mAmAmGmGmCmGmUmGmC mAmAmGmAmUmCmC
I_2 (2′OMe RNA)	mCmGmGmCmGmGmUmUmCmAmCmUmCmCmAmUmCmUmCmA mUmCmAmCmCmGmCmCmG mAmCmUmUmGmCmC
I_3 (2′OMe RNA)	mCmGmUmGmCmCmCmAmAmCmCmAmCmUmCmAmCmCmAmC mCmAmGmGmGmCmAmCmG mAmGmUmGmAmGmA
I_4 (2′OMe RNA)	mGmGmAmUmCmUmU mGmCmAmCmGmCmCmUmUmAmUmUm AmUmUmAmUmCmUmCmUmUmGmGmCmGmUmGmC
sgRNA	GCUAAUGGUGGAAACACAGAGUUUUAGAGCUAGAAAUAGCA AGUUAAAAUAAGGCUAGUCCGUUAUCAACUUGAAAAAGUGG CACCGAGUCGGUGC

Table S7. Sequences of primers and plasmid used in this study, related to Fig. 3-5, S15 and S17 (The underline sequence represents the target site of sgRNA).

Strand Name	Sequence (from 5' to 3')
EGFP primers	Forward: GAAGAACGGCATCAAGGTG
	Reverse: CTGGGTGCTCAGGTAGTGGT
Alb primers	Forward: GCACAGAATCCTTGGTGAACAG
	Reverse: ATGGAAGGTGAATGTTTCAGCA
Ntcp primers	Forward: AGGGGGACATGAACCTCAG
	Reverse: AGGTCCCCATCATAGATCCC
Sox17 primers	Forward: GTGGACCGCACGGAATTTG
	Reverse: GGAGATTCACACCGGAGTCA
Ck8 primers	Forward: TCCTCAGGCAGCTATATGAAGAG
	Reverse: GGTTGGCAATATCCTCGTACTGT
β -Actin primers	Forward: CACCATTGGCAATGAGCGGTTC
	Reverse: AGGTCTTTGCGGATGTCCACGT
Aat primers	Forward: ATGCTGCCCAGAAGACAGATA
	Reverse: CTGAAGGCGAACTCAGCCA
Cyp2c9 primers	Forward: GCCACATGCCCTACACAGATG
	Reverse: TAATGTCACAGGTCACTGCATGG
Cyp3a4 primers	Forward: AGCCTGGTGCTCCTCTATCT
	Reverse: CCCTTATGGTAGGACAAAAT
Cyp1a1 primers	Forward: ACATGCTGACCCTGGGAAAG
	Reverse: GGTGTGGAGCCAATTCGGAT
Ugt1a1 primers	Forward: TTGTCTGGCTGTTCCCACTTA
	Reverse: GGTCCGTCAGCATGACATCA
Ttr primers	Forward: CGTGCATGTGTTCAGAAAGGCTG
	Reverse: CTCCTCAGTTGTGAGCCCATGC
pCMV-BFP-P2A-EGFP-SSA	ATGGTGTCTAAGGGCGAAGAGCTGATTAAGGAGAACATGC<br>ACATGAAGCTGTACATGGAGGGCACCGTGGACAACCATCA<br>CTTCAAGTGCACATCCGAGGGCGAAGGCAAGCCCTACGAG<br>GGCACCCAGACCATGAGAATCAAGGTGGTCGAGGGCGGC

CCTCTCCCCTTCGCCTTCGACATCCTGGCTACTAGCTTCCTC
TACGGCAGCAAGACCTTCATCAACCACACCCAGGGCATCC
CCGACTTCTTCAAGCAGTCCTTCCCTGAGGGCTTCACATGG
GAGAGAGTCACCACATACGAAGACGGGGGCGTGCTGACC
GCTACCCAGGACACCAGCCTCCAGGACGGCTGCCTCATCT
ACAACGTCAAGATCAGAGGGGTGAACTTCACATCCAACGG
CCCTGTGATGCAGAAGAAAACACTCGGCTGGGAGGCCTTC
ACCGAGACGCTGTACCCCGCTGACGGCGGCCTGGAAGGCA
GAAACGACATGGCCCTGAAGCTCGTGGGCGGGAGCCATCT
GATCGCAAACGCCAAGACCACATATAGATCCAAGAAACCC
GCTAAGAACCTCAAGATGCCTGGCGTCTACTATGTGGACTA
CAGACTGGAAAGAATCAAGGAGGCCAACAACGAGACCTA
CGTCGAGCAGCACGAGGTGGCAGTGGCCAGATACTGCGAC
CTCCCTAGCAAACTGGGGCACAAGCTTAATGCTACTAACTT
CAGCCTGCTGAAGCAGGCTGGAGACGTGGAGGAGAACCC
TGGACCTATGGTGAGCAAGGGCGAGGAGCTGTTCACCGGG
GTGGTGCCCATCCTGGTCGAGCTGGACGGCGACGTAAACG
GCCACAAGTTCAGCGTGTCCGGCGAGGGCGAGGGCGATG
CCACCTACGGCAAGCTGACCCTGAAGTTCATCTGCACCAC
CGGCAAGCTGCCCGTGCCCTGGCCCACCCTCGTGACCACC
CTGACCTATGGAGTGCAGTGCTTCAGCCGCTACCCCGACC
ACATGAAGCAGCACGACTTCTTCAAGTCCGCCATGCCCGA
AGGCTACGTCCAGGAGCGCACCATCTTCTTCAAGGACGAC
GGCAACTACAAGACCCGCGCCGAGGTGAAGTTCGAGGGC
GACACCCTGGTGAACCGCATCGAGCTGAAGGGCATCGACT
TCAAGGAGGACGGCAACATCCTGGGGCACAAGCTGGAGT
ACAACTACAACAGCCACAACGTCTATATCATGTAAAAGCTaa
tggtggaaacacagaaggtaAATTCCCATGCCCGAAGGCTACGTCCAG
GAGCGCACCATCTTCTTCAAGGACGACGGCAACTACAAGA
CCCGCGCCGAGGTGAAGTTCGAGGGCGACACCCTGGTGA
ACCGCATCGAGCTGAAGGGCATCGACTTCAAGGAGGACGG

CAACATCCTGGGGCACAAGCTGGAGTACAACTACAACAGC
CACAACGTCTATATCATGGCCGACAAGCAGAAGAACGGCA
TCAAGGTGAACTTCAAGATCCGCCACAACATCGAGGACGG
CAGCGTGCAGCTCGCCGACCACTACCAGCAGAACACCCCC
ATCGGCGACGGCCCCGTGCTGCTGCCCGACAACCACTACC
TGAGCACCCAGTCCGCCCTGAGCAAAGACCCCAACGAGA
AGCGCGATCACATGGTCCTGCTGGAGTTCGTGACCGCCGC
CGGGATCACTCTCGGCATGGACGAGCTGTACAAGTCTGGT
GGTTCTCCCAAGAAGAAGAGGAAAGTCTAA

Table S8. Sequences of oligonucleotide targets used in this study, related to Fig. 3-4 and Fig. S15.

Strand Name	Sequence 1 (from 5' to 3')	Sequence 2 (from 5' to 3')	mRNA recognition (base number)
EGFP target	CAGGAGCGCACCATC TTCTTCAAGGACGA	ACTACCTGAGCACCCA GTCCGCCCTGAGC	283-311; 664-692
Ck8 target	GGAGCAGATCAAGA CCCTCAACAACAAGT	ACCAGATCAAGTATGA GGAGCTGCAGAGC	380-408; 949-977
Sox17 target	GACCCGGCTTTCTTC GCCGCCCCGATGCC	GCGGTATATTACTGCAA CTATCCTGACGT	901-929; 1417-1445
Alb target	GCAACACAAAGATG ACAACCCAAACCTCC	CCATGCAGATATATGCA CACTTTCTGAGA	422-450; 1640-1668
Cyp3a4 target	CTATAGCTGAGGATG AAGAATGGAAGAGA	CAGGCAAGCCTGTCAC CTTGAAAGACGTC	459-487; 600-628

REFERENCES AND NOTES

1. S. Yang, B. W. Bögels, F. Wang, C. Xu, H. Dou, S. Mann, C. Fan, T. F. de Greef, DNA as a universal chemical substrate for computing and data storage. *Nat. Rev. Chem.* **8**, 179–194 (2024).

2. L. M. Adleman, Molecular computation of solutions to combinatorial problems. *Science* **266**, 1021–1024 (1994).

3. D. Y. Zhang, G. Seelig, Dynamic DNA nanotechnology using strand-displacement reactions. *Nat. Chem.* **3**, 103–113 (2011).

4. J. Li, A. A. Green, H. Yan, C. Fan, Engineering nucleic acid structures for programmable molecular circuitry and intracellular biocomputation. *Nat. Chem.* **9**, 1056–1067 (2017).

5. G. M. Church, Y. Gao, S. Kosuri, Next-generation digital information storage in DNA. *Science* **337**, 1628–1628 (2012).

6. B. Wang, S. S. Wang, C. Chalk, A. D. Ellington, D. Soloveichik, Parallel molecular computation on digital data stored in DNA. *Proc. Natl. Acad. Sci. U.S.A.* **120**, e2217330120 (2023).

7. B. Yurke, A. J. Turberfield, A. P. Mills Jr., F. C. Simmel, J. L. Neumann, A DNA-fuelled molecular machine made of DNA. *Nature* **406**, 605–608 (2000).

8. G. Seelig, D. Soloveichik, D. Y. Zhang, E. Winfree, Enzyme-free nucleic acid logic circuits. *Science* **314**, 1585–1588 (2006).

9. D. Y. Zhang, E. Winfree, Control of DNA strand displacement kinetics using toehold exchange. *J. Am. Chem. Soc.* **131**, 17303–17314 (2009).

10. A. J. Genot, D. Y. Zhang, J. Bath, A. J. Turberfield, Remote toehold: A mechanism for flexible control of DNA hybridization kinetics. *J. Am. Chem. Soc.* **133**, 2177–2182 (2011).

11. W. Lai, L. Ren, Q. Tang, X. Qu, J. Li, L. Wang, L. Li, C. Fan, H. Pei, Programming chemical reaction networks using intramolecular conformational motions of DNA. *ACS Nano* **12**, 7093–7099 (2018).

12. L. Qian, E. Winfree, Scaling up digital circuit computation with DNA strand displacement cascades. *Science* **332**, 1196–1201 (2011).

13. F. Wang, H. Lv, Q. Li, J. Li, X. Zhang, J. Shi, L. Wang, C. Fan, Implementing digital computing with DNA-based switching circuits. *Nat. Commun.* **11**, 121 (2020).

14. X. Xiong, M. Xiao, W. Lai, L. Li, C. Fan, H. Pei, Optochemical control of DNA-switching circuits for logic and probabilistic computation. *Angew. Chem. Int. Ed. Engl.* **60**, 3397–3401 (2021).

15. Q. Tang, W. Lai, P. Wang, X. Xiong, M. Xiao, L. Li, C. Fan, H. Pei, Multi-mode reconfigurable DNA-based chemical reaction circuits for soft matter computing and control. *Angew. Chem. Int. Ed. Engl.* **60**, 15013–15019 (2021).

16. C. Zhang, X. Ma, X. Zheng, Y. Ke, K. Chen, D. Liu, Z. Lu, J. Yang, H. Yan, Programmable allosteric DNA regulations for molecular networks and nanomachines. *Sci. Adv.* **8**, eabl4589 (2022).

17. H. Lv, N. Xie, M. Li, M. Dong, C. Sun, Q. Zhang, L. Zhao, J. Li, X. Zuo, H. Chen, F. Wang, C. Fan, DNA-based programmable gate arrays for general-purpose DNA computing. *Nature* **622**, 292–300 (2023).

18. N. Srinivas, J. Parkin, G. Seelig, E. Winfree, D. Soloveichik, Enzyme-free nucleic acid dynamical systems. *Science* **358**, eaal2052 (2017).

19. N. Lobato-Dauzier, A. Baccouche, G. Gines, T. Lévi, Y. Rondelez, T. Fujii, S. H. Kim, N. Aubert-Kato, A. J. Genot, Neural coding of temperature with a DNA-based spiking chemical neuron. *Nat. Chem. Eng.* **1**, 510–521 (2024).

20. A. Padirac, T. Fujii, Y. Rondelez, Bottom-up construction of in vitro switchable memories. *Proc. Natl. Acad. Sci. U.S.A.* **109**, E3212–E3220 (2012).

21. A. J. Genot, A. Baccouche, R. Sieskind, N. Aubert-Kato, N. Bredeche, J. Bartolo, V. Taly, T. Fujii, Y. Rondelez, High-resolution mapping of bifurcations in nonlinear biochemical circuits. *Nat. Chem.* **8**, 760–767 (2016).

22. K. M. Cherry, L. Qian, Scaling up molecular pattern recognition with DNA-based winner-take-all neural networks. *Nature* **559**, 370–376 (2018).

23. L. Qian, E. Winfree, J. Bruck, Neural network computation with DNA strand displacement cascades. *Nature* **475**, 368–372 (2011).

24. X. Xiong, T. Zhu, Y. Zhu, M. Cao, J. Xiao, L. Li, F. Wang, C. Fan, H. Pei, Molecular convolutional neural networks with DNA regulatory circuits. *Nat. Mach. Intell.* **4**, 625–635 (2022).

25. S. Okumura, G. Gines, N. Lobato-Dauzier, A. Baccouche, R. Deteix, T. Fujii, Y. Rondelez, A. J. Genot, Nonlinear decision-making with enzymatic neural networks. *Nature* **610**, 496–501 (2022).

26. R. T. Nagipogu, D. Fu, J. H. Reif, A survey on molecular-scale learning systems with relevance to DNA computing. *Nanoscale* **15**, 7676–7694 (2023).

27. Y. J. Chen, B. Groves, R. A. Muscat, G. Seelig, DNA nanotechnology from the test tube to the cell. *Nat. Nanotech.* **10**, 748–760 (2015).

28. C. Zhang, Y. Zhao, X. Xu, R. Xu, H. Li, X. Teng, Y. Du, Y. Miao, H. C. Lin, D. Han, Cancer diagnosis with DNA molecular computation. *Nat. Nanotech.* **15**, 709–715 (2020).

29. Q. Ma, M. Zhang, C. Zhang, X. Teng, L. Yang, Y. Tian, J. Wang, D. Han, W. Tan, An automated DNA computing platform for rapid etiological diagnostics. *Sci. Adv.* **8**, eade0453 (2022).

30. R. Lopez, R. Wang, G. Seelig, A molecular multi-gene classifier for disease diagnostics. *Nat. Chem.* **10**, 746–754 (2018).

31. L. Yang, Q. Tang, M. Zhang, Y. Tian, X. Chen, R. Xu, Q. Ma, P. Guo, C. Zhang, D. Han, A spatially localized DNA linear classifier for cancer diagnosis. *Nat. Commun.* **15**, 4583 (2024).

32. S. M. Douglas, I. Bachelet, G. M. Church, A logic-gated nanorobot for targeted transport of molecular payloads. *Science* **335**, 831–834 (2012).

33. J. Hemphill, A. Deiters, DNA computation in mammalian cells: Microrna logic operations. *J. Am. Chem. Soc.* **135**, 10512–10518 (2013).

34. H. Pei, L. Liang, G. Yao, J. Li, Q. Huang, C. Fan, Reconfigurable three-dimensional DNA nanostructures for the construction of intracellular logic sensors. *Angew. Chem. Int. Ed. Engl.* **51**, 9020–9024 (2012).

35. H. Wang, P. Peng, Q. Wang, Y. Du, Z. Tian, T. Li, Environment-recognizing DNA-computation circuits for the intracellular transport of molecular payloads for mRNA imaging. *Angew. Chem. Int. Ed. Engl.* **59**, 6099–6107 (2020).

36. W. Wang, S. Huang, J. Li, K. Rui, S. Bi, J. R. Zhang, J. J. Zhu, Evaluation of intracellular telomerase activity through cascade DNA logic gates. *Chem. Sci.* **8**, 174–180 (2017).

37. M. Bai, F. Chen, X. Cao, Y. Zhao, J. Xue, X. Yu, C. Fan, Y. Zhao, Intracellular entropy-driven multi-bit DNA computing for tumor progression discrimination. *Angew. Chem. Int. Ed. Engl.* **59**, 13267–13272 (2020).

38. H. M. T. Choi, J. Y. Chang, L. A. Trinh, J. E. Padilla, S. E. Fraser, N. A. Pierce, Programmable in situ amplification for multiplexed imaging of mRNA expression. *Nat. Biotechnol.* **28**, 1208–1212 (2010).

39. L. Chen, W. Chen, G. Liu, J. Li, C. Lu, J. Li, W. Tan, H. Yang, Nucleic acid-based molecular computation heads towards cellular applications. *Chem. Soc. Rev.* **50**, 12551–12575 (2021).

40. B. Groves, Y. J. Chen, C. Zurla, S. Pochekailov, J. L. Kirschman, P. J. Santangelo, G. Seelig, Computing in mammalian cells with nucleic acid strand exchange. *Nat. Nanotech.* **11**, 287–294 (2016).

41. N. L. Dabby, H. L. Chen, J. M. Schaeffer, E. Winfree, "The kinetics of toehold-mediated four-way branch migration. Synthetic molecular machines for active self-assembly: Prototype algorithms, designs, and experimental study," thesis, California Institute of Technology, Pasadena, CA (2013).

42. D. Y. Duose, R. M. Schweller, J. Zimak, A. R. Rogers, W. N. Hittelman, M. R. Diehl, Configuring robust DNA strand displacement reactions for in situ molecular analyses. *Nucleic Acids Res.* **40**, 3289–3298 (2012).

43. S. X. Chen, D. Y. Zhang, G. Seelig, Conditionally fluorescent molecular probes for detecting single base changes in double-stranded DNA. *Nat. Chem.* **5**, 782–789 (2013).

44. M. Karymov, D. Daniel, O. F. Sankey, Y. L. Lyubchenko, Holliday junction dynamics and branch migration: Single-molecule analysis. *Proc. Natl. Acad. Sci. U.S.A.* **102**, 8186–8191 (2005).

45. F. Smith, A. Sengar, G. B. V. Stan, T. E. Ouldridge, M. Stevens, J. Goertz, W. Bae, Overcoming the speed limit of four-way DNA branch migration with bulges in toeholds. bioRxiv 2023.05.15.540824 [Preprint] (2023). https://doi.org/10.1101/2023.05.15.540824.

46. S. Venkataraman, R. M. Dirks, P. W. K. Rothemund, E. Winfree, N. A. Pierce, An autonomous polymerization motor powered by DNA hybridization. *Nat. Nanotech.* **2**, 490–494 (2007).

47. P. J. Santangelo, Molecular beacons and related probes for intracellular RNA imaging. *Wiley Interdiscip. Rev. Nanomed. Nanobiotechnol.* **2**, 11–19 (2010).

48. J. B. Bramsen, M. B. Laursen, A. F. Nielsen, T. B. Hansen, C. Bus, N. Langkjær, B. R. Babu, T. Højland, M. Abramov, A. V. Aerschot, D. Odadzic, R. Smicius, J. Haas, C. Andree, J. Barman, M. Wenska, P. Srivastava, C. Zhou, D. Honcharenko, S. Hess, E. Müller, G. V. Bobkov, S. N. Mikhailov, E. Fava, T. F. Meyer, J. Chattopadhyaya, M. Zerial, J. W. Engels, P. Herdewijn, J. Wengel, J. Kjems, A large-scale chemical modification screen identifies design rules to generate siRNAs with high activity, high stability and low toxicity. *Nucleic Acids Res.* **37**, 2867–2881 (2009).

49. D. P. Bratu, B. J. Cha, M. M. Mhlanga, F. R. Kramer, S. Tyagi, Visualizing the distribution and transport of mRNAs in living cells. *Proc. Natl. Acad. Sci. U.S.A.* **100**, 13308–13313 (2003).

50. S. F. Altschul, W. Gish, W. Miller, E. W. Myers, D. J. Lipman, Basic local alignment search tool. *J. Mol. Biol.* **215**, 403–410 (1990).

51. P. Huang, L. Zhang, Y. Gao, Z. He, D. Yao, Z. Wu, J. Cen, X. Chen, C. Liu, Y. Hu, D. Lai, Z. Hu, L. Chen, Y. Zhang, X. Cheng, X. Ma, G. Pan, X. Wang, L. Hui, Direct reprogramming of human fibroblasts to functional and expandable hepatocytes. *Cell Stem Cell* **14**, 370–384 (2014).

52. K. T. Lim, S. C. Lee, Y. Gao, K. P. Kim, G. Song, S. Y. An, K. Adachi, Y. J. Jang, J. Kim, K. J. Oh, T. H. Kwak, S. I. Hwang, J. S. You, K. Ko, S. H. Koo, A. D. Sharma, J. H. Kim, L. Hui, T. Cantz, H. R. Schöler, D. W. Han, Small molecules facilitate single factor-mediated hepatic reprogramming. *Cell Rep.* **15**, 814–829 (2016).

53. S. Ji, L. Zhang, L. Hui, Cell fate conversion: Direct induction of hepatocyte-like cells from fibroblasts. *J. Cell. Biochem.* **114**, 256–265 (2013).

54. S. P. Harrison, R. Siller, Y. Tanaka, M. E. Chollet, M. E. de la Morena-Barrio, Y. Xiang, B. Patterson, E. Andersen, C. Bravo-Pérez, H. Kempf, K. S. Åsrud, O. Lunov, A. Dejneka, M. C. Mowinckel, B. Stavik, P. M. Sandset, E. Melum, S. Baumgarten, F. Bonanini, D. Kurek, S. Mathapati, R. Almaas, K. Sharma, S. R. Wilson, F. S. Skottvoll, I. C. Boger, I. L. Bogen, T. A. Nyman, J. J. Wu, A. Bezrouk, D. Cizkova, J. Corral, J. Mokry, R. Zweigerdt, I. H. Park, G. J. Sullivan, Scalable production of tissue-like vascularized liver organoids from human PSCs. *Exp. Mol. Med.* **55**, 2005–2024 (2023).

55. R. Barrangou, J. A. Doudna, Applications of CRISPR technologies in research and beyond. *Nat. Biotechnol.* **34**, 933–941 (2016).

56. H. Li, Y. Yang, W. Hong, M. Huang, M. Wu, X. Zhao, Applications of genome editing technology in the targeted therapy of human diseases: Mechanisms, advances and prospects. *Sig. Transduct. Target. Ther.* **5**, 1 (2020).

57. M. Adli, The CRISPR tool kit for genome editing and beyond. *Nat. Commun.* **9**, 1911 (2018).

58. J. N. Zadeh, C. D. Steenberg, J. S. Bois, B. R. Wolfe, M. B. Pierce, A. R. Khan, R. M. Dirks, N. A. Pierce, NUPACK: Analysis and design of nucleic acid systems. *J. Comput. Chem.* **32**, 170–173 (2011).

59. Y. Yang, S. Liu, Y. Cheng, L. Nie, C. Lv, G. Wang, Y. Zhang, L. Hao, Highly efficient and rapid detection of the cleavage activity of Cas9/gRNA via a fluorescent reporter. *Appl. Biochem. Biotechnol.* **180**, 655–667 (2016).

60. S. C. Shih, G. S. Robinson, C. A. Perruzzi, A. Calvo, K. Desai, J. E. Green, I. U. Ali, L. E. Smith, D. R. Senger, Molecular profiling of angiogenesis markers. *Am. J. Pathol.* **161**, 35–41 (2002).

61. S. C. Shih, L. E. Smith, Quantitative multi-gene transcriptional profiling using real-time PCR with a master template. *Exp. Mol. Pathol.* **79**, 14–22 (2005).
